# Mitochondrial Targeting of the Enteropathogenic Escherichia coli Map Triggers Calcium Mobilization, ADAM10-MAP Kinase Signaling, and Host Cell Apoptosis

**DOI:** 10.1128/mBio.01397-20

**Published:** 2020-09-15

**Authors:** Rachana Pattani Ramachandran, Chaya Spiegel, Yael Keren, Tsafi Danieli, Naomi Melamed-Book, Ritesh Ranjan Pal, Efrat Zlotkin-Rivkin, Ilan Rosenshine, Benjamin Aroeti

**Affiliations:** a Department of Cell and Developmental Biology, Alexander Silberman Institute of Life Sciences, The Hebrew University of Jerusalem, Jerusalem, Israel; b The Protein Production Facility, Wolfson Centre for Applied Structural Biology, Alexander Silberman Institute of Life Sciences, The Hebrew University of Jerusalem, Jerusalem, Israel; c Bioimaging Unit, Alexander Silberman Institute of Life Sciences, The Hebrew University of Jerusalem, Jerusalem, Israel; d Department of Microbiology and Molecular Genetics, Institute for Medical Research Israel-Canada, Faculty of Medicine, The Hebrew University of Jerusalem, Jerusalem, Israel; University of Illinois Urbana; UCLA School of Medicine

**Keywords:** ADAM10, calcium, enteropathogenic *E. coli*, MAP kinases, Map effector, protein effectors, apoptosis, mitochondria

## Abstract

The ability of diarrheagenic bacterial pathogens, such as enteropathogenic Escherichia coli (EPEC), to modulate the activity of mitogen-activated protein kinases (MAPKs) and cell survival has been suggested to benefit bacterial colonization and infection. However, our understanding of the mechanisms by which EPEC modulate these functions is incomplete. In this study, we show that the EPEC type III secreted effector Map stimulates the sheddase activity of the disintegrin and metalloproteinase domain-containing protein 10 (ADAM10) and the ERK and p38 MAPK signaling cascades. Remarkably, all these activities were dependent upon the ability of Map to target host mitochondria, mainly via its mitochondrial toxicity region (MTR). Map targeting of mitochondria disrupted the mitochondrial membrane potential, causing extrusion of mitochondrial Ca^2+^ into the host cell cytoplasm. We also found that Map targeting of mitochondria is essential for triggering host cell apoptosis. Based on these findings, we propose a model whereby Map imported into mitochondria causes mitochondrial dysfunction and Ca^2+^ efflux into the host cytoplasm. Since Ca^2+^ has been reported to promote ADAM10 activation, the acute elevation of Ca^2+^ in the cytoplasm may stimulate the ADAM10 sheddase activity, resulting in the release of epidermal growth factors that stimulate the ERK signaling cascade. As p38 activity is also Ca^2+^ sensitive, elevation in cytoplasmic Ca^2+^ may independently also activate p38. We hypothesize that Map-dependent MAPK activation, combined with Map-mediated mitochondrial dysfunction, evokes mitochondrial host cell apoptosis, potentially contributing to EPEC colonization and infection of the gut.

## Abstract (precis)

Enteropathogenic E. coli (EPEC) is an important human diarrhea-causing bacterium. The pathogenic effects of EPEC largely depend upon its ability to inject a series of proteins, termed effectors, into the host cells. One such effector is the mitochondrion-associated protein (Map). Map has been shown to induce actin-rich projections (i.e., filopodia) on the infected cell surface and activate a Rho GTPase enzyme termed Cdc42. Nonetheless, although most injected Map localizes to host mitochondria, its functions in the mitochondria remain unknown. Here, we show that Map targeting of mitochondria stimulates the disruption of mitochondrial membrane potential to induce Ca^2+^ efflux into the host cytoplasm. The efflux stimulates the activity of a protein termed ADAM10, which induces activation of a mitogen-activated protein kinase cascade leading to host cell apoptosis. As apoptosis plays a central role in host-pathogen interactions, our findings provide novel insights into the functions of mitochondrial Map in promoting the EPEC disease.

## INTRODUCTION

Enteropathogenic Escherichia coli (EPEC) is a human-specific bacterial pathogen that infects the enterocytes of the small intestine. EPEC infection causes acute and persistent diarrhea, mainly in children worldwide (1, 2). The virulence of EPEC is primarily due to the ability of the microbe to activate a type III secretion system (T3SS) that injects dozens of effector proteins from the bacterial cytoplasm into the host cells (3). The translocated effectors intoxicate the infected cells by hijacking and subverting diverse organelles, cytoskeletal elements, and signaling processes (4, 5). Analysis of the precise mechanisms by which these effectors perform their functions is crucial for better understanding the EPEC disease and for designing improved therapeutics.

Mitogen-activated protein kinases (MAPKs) are involved in the regulation of cell proliferation, survival, differentiation, stress response, and programmed cell death (i.e., apoptosis) (6–8). We recently showed that EspH, an EPEC type III secreted effector implicated in actin cytoskeleton remodeling (9–11) and the inhibition of Rho GTPases (10, 12), also suppresses the MAPK/extracellular signal-regulated kinases 1/2 (ERK1/2) signaling pathway at longer infection times (13). Previous studies have indicated that EPEC can rapidly stimulate the MAPK/ERK1/2 signal transduction pathway and that this T3SS-dependent event may play a role in the inflammatory response and infection, but not in tight-junction barrier disruption (14–16). However, the identity and mode of action of type III secreted components that mediate ERK1/2 activation have not been explored. Here, we provide evidence that the type III secreted effector protein mitochondrion-associated protein (Map) activates the MAPK/ERK1/2 signaling pathway at an early infection phase. Map has been previously characterized to target mitochondria by a mitochondrial targeting signal (MTS) (17, 18), activate the Rho GTPase Cdc42 by a WxxxE guanine nucleotide exchange factor (GEF) family motif (19, 20), and interact with host proteins through a C-terminal TRL PDZ class I binding motif (21). Here, we show that Map stimulates the MAPK/ERK signaling pathway by activating the sheddase activity of the disintegrin and metalloproteinase domain-containing protein 10 (ADAM10). We linked these effects to the ability of Map to target mitochondria and evoke Ca^2+^ efflux from them into the host cell cytoplasm and to the induction of host apoptosis. We hypothesize that the triggering of the ADAM10-MAPK/ERK signaling by Map in an early infection event, which is counteracted by EspH in a later infection time, may play a crucial role in the induction of EPEC pathogenesis.

## RESULTS

### T3SS-dependent increase in pERK levels at an early infection stage.

**FIG 1:**
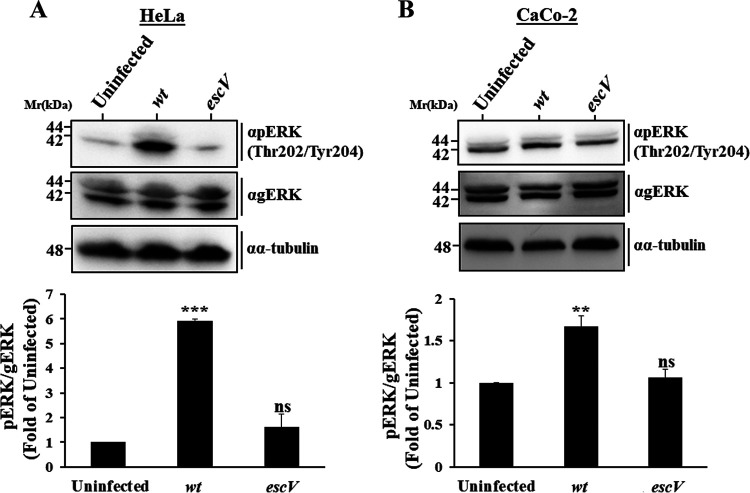
EPEC infection increases pERK levels at an early infection phase and in a T3SS-dependent manner. pERK levels were examined in HeLa (A) and Caco-2 (B) cells. Cells were infected with the indicated EPEC strains (Table S1) for 30 min at 37°C or remained uninfected. Cells were then lysed, and pERK and general (g)ERK levels were determined by SDS-PAGE followed by Western blotting, as described in Materials and Methods. Representative Western blots are shown. Results are means and SE from 3 independent experiments. ***, *P* < 0.0005; **, *P* < 0.005; ns, nonsignificant (*P* > 0.05).

We showed previously that active phosphorylated ERK (pERK) levels are suppressed upon prolonged (90-min) infection with EPEC and that the EspH effector mediates this effect (13). Here, we examined the effect that EPEC infection at a shorter time (30 min) may have on ERK phosphorylation (pERK). HeLa (Fig. 1A) or Caco-2 (Fig. 1B) cells were infected with EPEC-wt or with the type III secretion-deficient mutant EPEC-*escV* for 30 min at 37°C or were left uninfected. Cells were then lysed, and the levels of pERK were evaluated by immunoblotting. The results show a significant increase in pERK levels (mainly pERK2) in response to EPEC-wt compared to EPEC-*escV*-infected or uninfected cells. Infection with EPEC-*escV* had no significant impact on pERK levels compared to uninfected cells. These data suggest that type III secreted components increase pERK levels in epithelial cells at an early infection phase.

10.1128/mBio.01397-20.7 TABLE S1 Bacterial EPEC strains (all mutant strains are derivatives of E2348/69). Download Table S1, DOCX file, 0.02 MB. Copyright © 2020 Ramachandran et al. 2020 Ramachandran et al. This content is distributed under the terms of the Creative Commons Attribution 4.0 International license.

### Translocated Map induces ERK phosphorylation.

**FIG 2:**
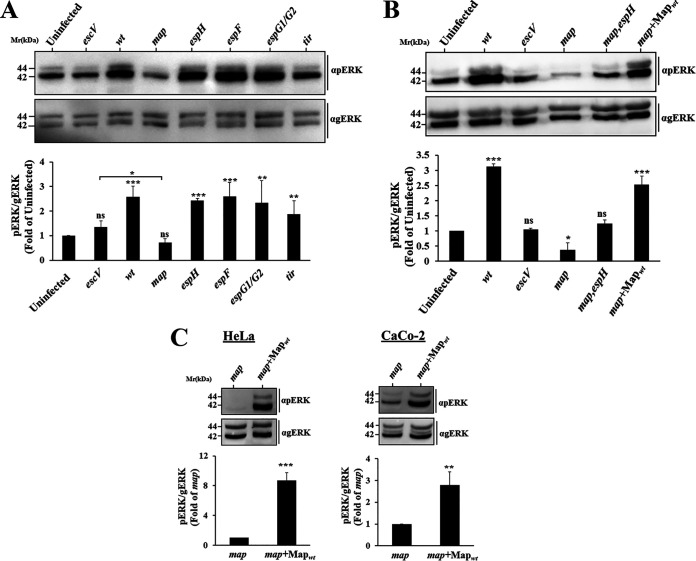
The type III secreted effector Map triggers an increase in pERK levels in HeLa and Caco-2 cells. (A) Screening for effects on pERK levels by EPEC strains mutated at different LEE-borne effector genes. HeLa cells were infected with the indicated mutant strains (Table S1), and pERK levels were analyzed, as for Fig. 1. (B and C) Map_wt_ translocation induces pERK in HeLa and CaCo-2 cells, and EspH counteracts this effect. Cells were infected with the indicated EPEC strains (Table S1), and pERK levels in the infected cells were analyzed, as for Fig. 1. Results are means and SE from 3 independent experiments. ***, *P* < 0.0005; **, *P* < 0.005; *, *P* < 0.05; ns, nonsignificant (*P* > 0.05).

Our next goal was to identify type III secreted effectors that could mediate the stimulatory effect. To this end, we examined the capacity of EPEC strains mutated at the different locus of enterocyte effacement (LEE)-located effector genes to upregulate pERK in HeLa cells. The results identified EPEC-*map* as the only mutant strain that did not demonstrate such capability (Fig. 2A). Notably, pERK levels in EPEC-*map*-infected cells were even lower than those exhibited by EPEC-*escV*-infected cells. This finding suggests that in the absence of Map, other bacterial components suppress ERK. We suspected that the injected EspH, a negative regulator of ERK (13), could be such a component. To address this prediction, pERK levels in HeLa cells infected with an EPEC strain doubly mutated in the *espH* and *map* genes (EPEC-*map*,*espH*) were examined. Indeed, data presented in Fig. 2B shows that pErK levels in cells infected with the EPEC-*map*,*espH* mutant strain were higher than in the EPEC-*map-*infected cells, reaching levels comparable to those exhibited by EPEC-*escV*-infected or uninfected cells. These results point to EspH as the effector that suppresses pERK in the EPEC-*map*-infected cells. Complementing EPEC-*map* with a Map-encoding plasmid increased pERK levels, which reached the levels observed in EPEC-wt-infected cells (Fig. 2B). A similar Map-dependent stimulatory effect, albeit somewhat lower, was observed in infected Caco-2 cells (Fig. 2C). Notably, in these experiments, Map was effectively translocated into the cells (Fig. S1A). Since the host actin cytoskeleton can be affected by bacterial targeted MAPKs (22), and since Map itself can modulate filamentous actin (F-actin) (10, 19, 23), the F-actin levels in EPEC-*map*+Map infection sites were compared to those of EPEC-*map*-infected cells. The results showed that the F-actin clustering levels were comparable at the infection sites of the two EPEC strains (Fig. S1B). These results argue that translocated Map is the type III secreted effector that confers pERK stimulation at an early infection time and that the effect is not contributed by changes in F-actin clustering at infection sites.

10.1128/mBio.01397-20.1 FIG S1 (A) Map_wt_ is translocated into infected host cells. HeLa cells were infected with the indicated EPEC strains for 30 min at 37°C and the effector translocation assay was applied, as described in Materials and Methods. Cells were lysed in NP-40-containing buffer, and the detergent-soluble (containing host cell-associated effector proteins) and insoluble (containing bacteria-associated effector proteins) fractions were isolated and subjected to Western blotting analyses; blots were probed with anti-HA antibodies for detecting the HA-tagged Map_wt_ in the two fractions and with anti-β-actin antibodies for detecting total protein levels in the lysate. The results show the presence of Map_wt_ in the detergent-soluble fractions in EPEC-*map*+Map_wt_- but not in EPEC-*escV*+Map_wt_-infected cells or uninfected cells. Map_wt_ was detected in the detergent-insoluble fractions of both EPEC-*map*+Map_wt_- and EPEC-*escV*+Map_wt_-infected cells, confirming that the bacterial protein was expressed in the infecting bacteria. The results suggest that Map_wt_ is translocated into the infected cells in a T3SS-dependent manner. (B) Translocated Map_wt_ does not affect F-actin clustering at infection sites. HeLa cells infected with EPEC-*map* or EPEC-*map*+Map_wt_ for 30 min at 37°C were washed, fixed and immunostained with anti-HA antibodies (for visualizing Map), followed by staining with Texas Red-phalloidin (for visualizing F-actin) and DAPI (for visualizing the DNA of the host cell nucleus and bacteria). Cells were imaged by confocal microscopy, and the fluorescence levels of F-actin associated with infecting bacterial microcolonies were analyzed, as described in Materials and Methods. Representative images are shown. Arrows indicate infecting microcolonies. Bar = 10 μm. The results show a lack of significant effect of translocated Map on F-actin clustering at infection sites. (C) Silencing of ADAM10 expression by siRNA. HeLa cells were transfected with ADAM10 siRNA and control siRNA (Table S4), as described in Materials and Methods. Untransfected cells served as controls. Cells were then lysed and subjected to Western blot analysis; blots were probed with anti-ADAM10 antibodies, and with anti-β-actin antibodies for estimating total protein levels. Band intensity was quantified. Results show about 80% reduction in ADAM10 protein level in the siRNA-treated cells, compared to untransfected cells. The ADAM10 protein level in the control siRNA-treated cells was similar to the level in untransfected cells. (D) Translocation of Map is not affected by silencing ADAM10 expression. The Map effector translocation assay was performed in ADAM10 siRNA and ADAM10 control-treated cells, as described in Materials and Methods. Western blots were probed with an anti-HA antibody for detecting Map, anti-ADAM10 antibody, and anti-β-actin antibodies. The results show a significantly reduced expression of ADAM10 in the ADAM10 siRNA compared to control siRNA-treated cells, confirming the efficacious knockdown of ADAM10 expression in the siRNA-treated cells. Map levels in the detergent-soluble fraction of the ADAM10 siRNA-treated cells were comparable to those detected in the control siRNA, indicating that Map translocation into the infected host cells was not affected by silencing ADAM10 expression. (E) Map_wt_, but not Map_WxxxA_, activates Cdc42. HeLa cells were infected with the indicated Map strains (Table S1) for 30 min at 37°C. Active Cdc42 levels in the host cells were determined by a GST pulldown assay, as described in Materials and Methods. Active Cdc42 (precipitated [P] with GST-PBD) was detected by immunoblotting (IB) using anti-Cdc42 antibodies (upper gel). Total levels of immunoblotted Cdc42 and β-actin in the cell lysates are shown in the middle and bottom gels, respectively. The level of active Cdc42 in EPEC-*map*+Map- and EPEC-*map*+Map_WxxxA_ (Map mutated in the Rho GEF motif)-infected cells was determined by normalizing the active Cdc42 (i.e., Cdc42 pulled down by GST-PBD) levels to the total level of Cdc42 in the lysate. The value obtained was further normalized to active Cdc42 in EPEC-*map*-infected cells. (F to H) Ectopic expression of Map activates pERK. (F) Ectopic expression of Map. HeLa cells were transfected with eGFP, Map-eGFP, mCherry, or mCherry-Map (Table S3) for 15 h, as described in Materials and Methods. Cells were lysed in SDS-PAGE sample buffer, and protein expression was analyzed by Western blotting and probing with the indicated antibodies. Results show that Map-eGFP expression levels were lower than those of eGFP. (G) Effects of Map expression on pERK levels. The indicated Map-encoding constructs were expressed in HeLa cells, and their effects on pERK levels were analyzed by Western blotting, as described in Materials and Methods. The results show increased pERK levels in cells transfected with Map-eGFP compared to cells expressing eGFP alone. Expression of mCherry-Map did not affect pERK levels, compared to mCherry expression. This could be attributed to the fact that mCherry, which tagged the N terminus of Map, was juxtaposed to the N-terminally located MTS (Fig. 5A) and thus interfered with Map targeting of mitochondria. (H) Localization of ectopically expressed Map relative to mitochondria. HeLa cells transfected with the indicated Map-encoding plasmids were immunostained with anti-Hsp60 antibodies and stained with DAPI to visualize cell nuclei. Cells were then imaged by confocal microscopy. Representative images are shown. The results show that ectopically expressed Map-eGFP colocalized extensively with immunolabeled Hsp60, suggesting that the expressed protein targets mitochondria. In contrast, ectopically expressed mCherry-Map showed diffuse staining, which did not coincide with the Hsp60 labeling, suggesting that targeting of mitochondria by the Map protein was impaired. Bar = 5 μm. (I) Analysis of Map localization to mitochondria. HeLa cells were infected with EPEC-*map* or EPEC-*map*+Map_wt_ strains. Cells were subsequently immunostained with anti-HA (Map) and anti-Hsp60 (mitochondria) antibodies. Cells were also stained with DAPI for visualizing the DNA of the host nucleus and bacteria and imaged by confocal microscopy. Representative images are shown. White arrows indicate infecting EPEC microcolonies. Bar = 5 μm. Fluorescence intensity profiles were generated along a drawn line over Map- and mitochondrion-colabeled areas, as exemplified in the boxed area and the corresponding graph (bottom left). Green arrows indicate areas where the fluorescence of the two markers copeaked and is therefore considered colocalizing labeling. Red arrows indicate areas where this phenomenon was not observed and the fluorescence is therefore considered noncolocalizing labeling. The percent colocalization was determined and shown in the bar graph. (J to M) Map, but not EspF, predominantly stimulates pERK. (J) Effector translocation. Translocation assay was applied to estimate the translocation efficacy of Flag-tagged EspF expressed in the background of EPEC-*map* or EPEC-*map*,*espF* strains (Table S1), as described in Materials and Methods. The results show comparable EspF translocation levels upon HeLa cell infections with these strains. Infection with EPEC-*map* served as controls for antibody specificity. α-Tubulin was used as the loading control. (K and L) Effects on pERK. HeLa cells were infected with the indicated bacterial strains, and the effect of bacterial infection on pERK levels was determined by immunoblotting, as before. α-Tubulin was used as the loading control. Results are means and SE from 3 independent experiments. The results show that EspF is capable of stimulating pERK levels, but at lower levels than Map_wt_. (M) Localization of translocated EspF relative to mitochondria. HeLa cells were infected with the *map*+EspF_wt_ strain for 30 min at 37°C, and the cells were then immunostained with anti-Flag (EspF) and anti-Hsp60 (mitochondria) antibodies. These cells were also stained with DAPI for visualizing the DNA of the host nucleus and bacteria, respectively. Cells were analyzed by confocal microscopy and representative images are shown. The white arrows indicate infecting EPEC microcolonies. Bar = 5 μm. The box intensity profiles of EspF and Hsp60 are shown (bottom) and the degree of colocalization between the two proteins was determined as described in Materials and Methods and above (for panel I). The translocated EspF was mostly found to be located nearby the infection sites and only partially colocalizes with the mitochondria. Download FIG S1, PDF file, 1.6 MB. Copyright © 2020 Ramachandran et al. 2020 Ramachandran et al. This content is distributed under the terms of the Creative Commons Attribution 4.0 International license.

### Map triggers phosphorylation of MEK, p38, and the EGFR but not B-Raf or C-Raf.

**FIG 3:**
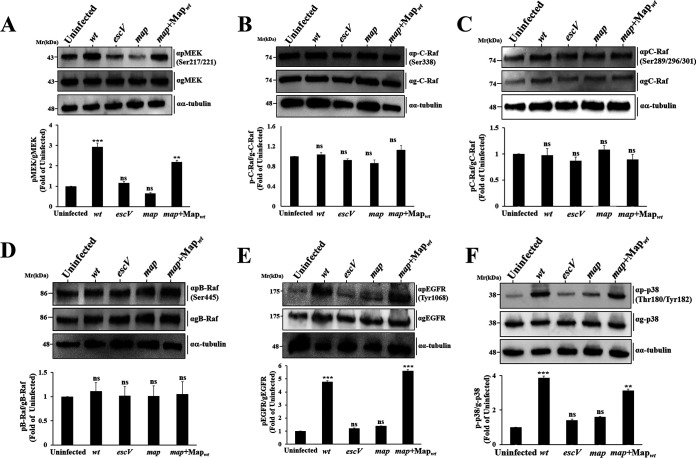
Map activates a MAPK signaling cascade upstream of ERK. HeLa cells were infected with wild-type, *escV*, *map*, or *map*+Map_wt_ EPEC strains for 30 min at 37°C. Cells were lysed and subjected to SDS-PAGE followed by Western blotting analyses and probed with the indicated anti-phosphorylated (p) and general (g) MEK (A), C-Raf (B–C), B-Raf (D), EGFR (E), and p38 (F) antibodies (Table S2). The levels of phosphorylated and total proteins were determined as described for Fig. 1. Results are means and SE from 3 independent experiments. Notably, we used three different anti-phospho-Raf antibodies to strengthen the conclusion that Map stimulates the EGFR-MAPK pathway in a Raf-independent mechanism. The anti-pRaf antibodies used to detect Raf kinase stimulation linked to the EGFR signaling were the classical anti-c-Raf phosphorylated on Ser338 and the anti-B-Raf phosphorylated on Ser445, a site in B-Raf shown to be equivalent to that in c-Raf (Ser338) (68), and anti-pc-Raf phosphorylated on Ser289, Ser296, and Ser301. These serine residues of c-Raf have been reported to be EGF induced (69). ***, *P* < 0.0005; **, *P* < 0.005; ns, nonsignificant (*P* > 0.05).

Next, we asked what the upstream components that activate ERK upon stimulation by Map are. We found that the immediate upstream MEK (Fig. 3A), but not C-Raf (Fig. 3B and C) or B-Raf (Fig. 3D), is hyperphosphorylated in response to EPEC-*map*+Map_wt_ infection. Interestingly, pEGFR (Y1068), positioned upstream of MEK, was also upregulated (Fig. 3E), suggesting that translocated Map activates the epidermal growth factor receptor (EGFR)/MEK/ERK signaling pathway, via a Raf-independent mechanism.

10.1128/mBio.01397-20.8 TABLE S2 Primary and secondary antibodies. Download Table S2, DOCX file, 0.01 MB. Copyright © 2020 Ramachandran et al. 2020 Ramachandran et al. This content is distributed under the terms of the Creative Commons Attribution 4.0 International license.

Map activates the Rho GTPase Cdc42 (19, 20). Cdc42 and Rac1 can act as upstream mediators of p38 and JNK MAPK activities in response to stress or inflammatory stimuli (24). Therefore, we hypothesized that Map may stimulate the phosphorylation and activation of p38. Data in Fig. 3F indeed show a Map-dependent increase in phosphorylated-p38 (p-p38) levels. pJNK was also stimulated in response to EPEC-wt, but the clear involvement of Map in the process could not be demonstrated (data not shown). In summary, our data link Map to the stimulation of pEGFR, pMEK, pERK, and p-p38 but not to pRaf signaling.

### Map mediates ADAM10 but not ADAM17 activation.

**FIG 4:**
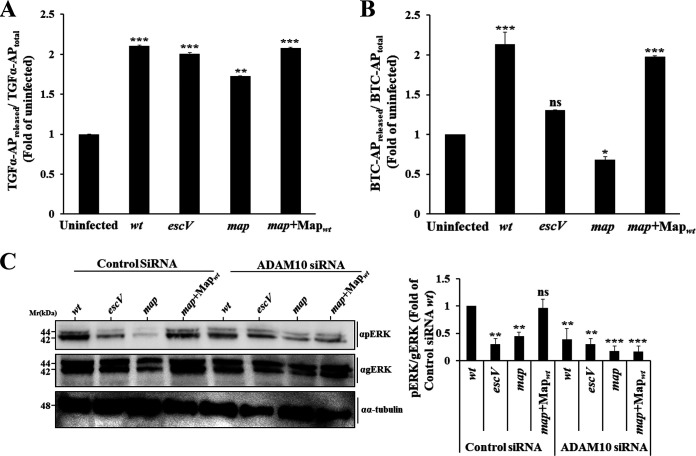
ADAM10, but not ADAM17, is involved in Map-dependent activation of pERK. (A and B) Map impact on ADAM17 and ADAM10 sheddase activity. HeLa cells were transfected with TGF-α–AP-encoding (for monitoring ADAM17) (A) or BTC-AP-encoding (for monitoring ADAM10) (B) plasmids. Cells were then infected with the indicated EPEC strains for 30 min at 37°C, and the levels of sheddase activity were determined as described in Materials and Methods. Each experiment was performed in triplicate, and results are means and SE from 3 independent experiments. (C) Effects of ADAM10 silencing on pERK levels. HeLa cells were transfected with either ADAM10 siRNA or control (scrambled) siRNA, as described in Materials and Methods. Cells were then infected with the indicated EPEC strains for 30 min at 37°C, lysed, and subjected to SDS-PAGE followed by Western blotting for determining pERK levels. Results are means and SE from 3 independent experiments. ***, *P* < 0.0005; **, *P* < 0.005; *, *P* < 0.05; ns, nonsignificant (*P* > 0.05).

ADAM17 acts as a sheddase of epiregulin, transforming growth factor α (TGF-α), amphiregulin, and heparin-binding EGF-like growth factor (25). Since these ligands are known to potentiate the EGFR/ERK/MAPK pathway (26), we reasoned that Map might activate this pathway by stimulating ADAM17. Data in Fig. 4A suggest that this is not the case. EPEC infection activates the ADAM17-mediated TGF-α sheddase activity, but the process is T3SS and Map independent. EGF and betacellulin (BTC) share overlapping signaling properties concerning EGFR activation (27). Because the metalloprotease ADAM10 acts as the main sheddase of BTC and EGF (25), we hypothesized that Map might be capable of activating the ADAM10 sheddase activity. Results presented in Fig. 4B show that cell infection with EPEC-wt or EPEC-*map*+Map_wt_ indeed resulted in increased BTC shedding compared to EPEC-*map* or EPEC-*escV* infection or no infection. These data suggest that Map stimulates the ADAM10 sheddase activity. To further confirm this conclusion, we examined the ability of EPEC to upregulate pERK in cells whose ADAM10 expression was silenced by small interfering RNA (siRNA) (Fig. S1C). Data in Fig. 4C show that cell infection with EPEC-*map*+Map_wt_ failed to increase pERK levels in the ADAM10 siRNA-treated cells, while the stimulatory effect was apparent in the control siRNA-treated cells. Notably, Map translocation was not affected by ADAM10 siRNA treatment (Fig. S1D). These results suggest that the Map-mediated stimulation of pERK is due to its ability to activate ADAM10.

### The GEF and PDZ binding motifs of Map are not involved in ADAM10-EGFR-MAPK activation.

**FIG 5:**
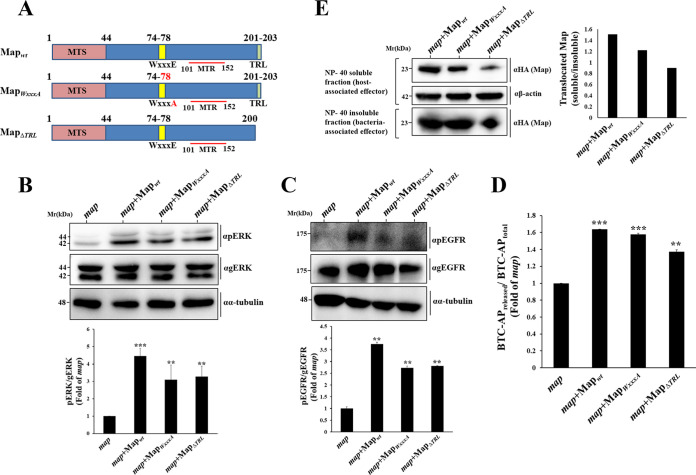
The Rho GEF or PDZ binding motifs of Map are not required for ADAM10-EGFR-MAPK activation. (A) Schematic presentation of wild-type Map (Map_wt_) and Map mutants (Map_WxxxA_ and Map_ΔTRL_) used in this study. The Map mitochondrial targeting signal (MTS; amino acids [aa] 1 to 44), the WxxxE Rho GEF domain (aa 74 to 78), the mitochondrial toxicity region (MTR, aa 101 to 152), and the C-terminal PDZ type I binding TRL motif (aa 201 to 203) are indicated. (B and C) Effects of translocated Map on pERK and pEGFR levels. HeLa cells were infected with the indicated EPEC strains, and the levels of pERK and pEGFR in the infected cells were determined, as described in Materials and Methods. Results are means and SE of 3 independent experiments. (D) Effects of MAP on ADAM10 sheddase activity. HeLa cells transfected with the BTC-AP encoding plasmid were infected with the indicated EPEC strains for 30 min at 37°C and the level of ADAM10 sheddase activity was determined as described in Materials and Methods. (E) Map translocation. HeLa cells were infected with the indicated EPEC strains and the Map translocation into the infected cells was assessed by the effector translocation assay, as described in Materials and Methods. ***, *P* < 0.0005; **, *P* < 0.005.

As previously noted, Map consists of an MTS, a WxxxE Rho GEF, and a C-terminal TRL class I PDZ binding motif. To investigate which of these motifs mediate ERK activation, we generated EPEC mutant strains bearing a mutation in the WxxxE or the TRL motif (Table S1 and Fig. 5A). The capacity of these strains to alter the pERK levels in infected HeLa cells was evaluated, as before. pERK levels in EPEC-*map*- and EPEC-*map*+Map_wt_-infected cells served as a reference in these experiments. Results show that infection with EPEC-*map*+Map_WxxxA_ and EPEC-*map*+Map_ΔTRL_ caused significant upregulation of pERK levels compared to those in EPEC-*map*-infected cells (Fig. 5B). A similar effect was observed on pMEK (data not shown), pEGFR (Fig. 5C), and ADAM10 BTC sheddase activity (Fig. 5D). Compared to Map_wt_, the capacity of the mutant Maps to undergo translocation into the host cells was somewhat reduced, especially in the case of Map*_Δ_*_TRL_ (Fig. 5E). This may explain the slightly reduced levels of pERK and pEGFR levels in cells infected with these EPEC strains compared to levels in cells infected with the MAP_wt_ strain. However, the amounts of the translocated effectors seemed to be sufficient for inducing the indicated effects. Notably, previous studies suggested that F-actin-rich filopodia are not generated in response to translocated Map*_Δ_*_TRL_ (19, 21). Map_WxxxA_ also fails to evoke Cdc42 and filopodium formation (Fig. S1E) (19). These data suggest that Map activates the ADAM10-MAPK and the Rho GTPase signaling pathways by two distinct and independent mechanisms.

Finally, in this context, ectopic expression of Map tagged in its C terminus with eGFP (Map-eGFP) (Fig. S1F) resulted in an increase in pERK levels compared to eGFP-expressing cells (Fig. S1G). The fusion protein colocalized with the mitochondrial marker Hsp60 (Fig. S1H). In contrast, ectopic expression of N-terminal mCherry-tagged Map did not colocalize with Hsp60 (Fig. S1H) and did not affect pERK levels (Fig. S1G) compared to mCherry expression. These results suggest that the expression of Map is sufficient for stimulating the ADAM10-MAPK signaling cascade and that targeting of mitochondria by the effector may play a role in the process.

### ADAM10 and MAPK activation requires mitochondrial targeting of Map.

**FIG 6:**
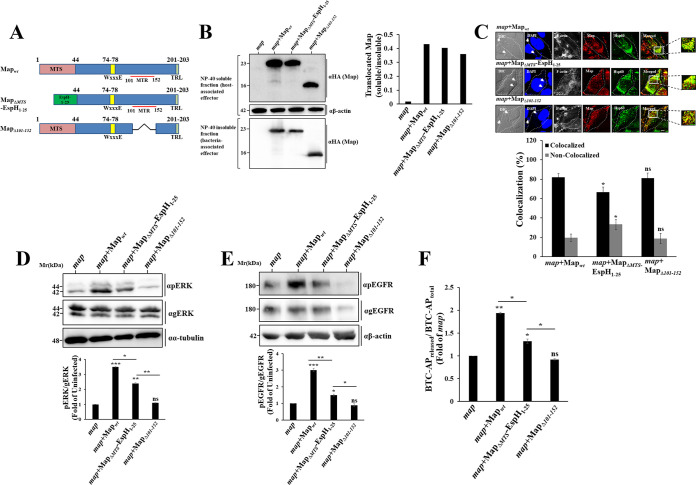
Map targeting of mitochondria is essential for ADAM10-EGFR-MAPK activation. (A) Schematic presentation of Map_wt_ and the Map_ΔMTS_-EspH_1–25_ and Map_Δ101–152_ mutants used in this study. (B) Map translocation. HeLa cells were infected with the indicated EPEC strains and the translocation assay was applied to evaluate Map translocation into the host cells, as described in Materials and Methods. (C) Map localization to mitochondria. HeLa cells were infected with the indicated EPEC strains, and cells were immunostained with anti-HA (Map) and anti-Hsp60 (mitochondria) antibodies. Cells were also stained with DAPI for visualizing the DNA of the host nucleus and bacteria, respectively. Cells were then analyzed by confocal microscopy, and representative images are shown. Arrows indicate infecting EPEC microcolonies. Bar = 5 μm. The degree of Map-mitochondrion colocalization was determined as described in Materials and Methods and the caption of Fig. S1I. (D and E) Effects of translocated Map on pERK and pEGFR levels. HeLa cells were infected with the indicated EPEC strains and the levels of pERK and pEGFR were determined, as before. Results are means and SE from 3 independent experiments. (F) Effects of translocated Map on ADAM10 sheddase activity. HeLa cells transfected with the BTC-AP-encoding plasmid were infected with the indicated EPEC strains and the level of ADAM10 sheddase activity was determined, as before. Results are means and SE from 3 independent experiments. ***, *P* < 0.0005; **, *P* < 0.005; *, *P* < 0.05; ns, nonsignificant (*P* > 0.05).

Next, we examined the importance of Map’s mitochondrial targeting in the upregulation of the ADAM10-pERK axis. The N-terminal ∼25-amino-acid sequences of bacterial type III effectors typically contain a signal that mediates their specific secretion via the T3SS (28). Thus, the MTS of Map, which was localized to the N-terminal 44-amino-acid segment of the protein (18), likely contains the signal that mediates its translocation via the T3SS. Therefore, the mere deletion of the MTS (Map_ΔMTS_) would not be a reasonable approach for examining its role in signaling, as such mutation may affect the translocation of the effector into the host. To address this potential problem, the N-terminal 25-amino-acid type III secretion signal of EspH, an effector whose localization upon translocation has been reported to be confined to the bacterial infection sites and not to mitochondria (9), was fused to the N terminus of Map_ΔMTS_ (Map_ΔMTS_-EspH_1–25_) (Tables S1 and S3; Fig. 6A). This modification was expected to facilitate the translocation of the MTS deleted Map mutant. Data presented in Fig. 6B indeed show that the translocation efficacy of the chimeric effector was comparable to that of Map_wt_. The translocated Map_ΔMTS_-EspH_1–25_ also showed a wide distribution within the infected cells, albeit with a somewhat reduced colocalization with the mitochondrial marker Hsp60, compared to Map_wt_ (Fig. 6C). Cell infection with EPEC-*map*+Map_ΔMTS_-EspH_1–25_ caused diminished pERK (Fig. 6D), pMEK (data not shown), pEGFR (Fig. 6E), and ADAM10 (Fig. 6F) activity, reaching intermediate values between those attained upon infection with EPEC-*map* and EPEC-*map*+Map_wt_. These results signify the importance of Map targeting of mitochondria in evoking the ADAM10-EGFR-MAPK signaling. Nevertheless, the observation that the majority of the chimeric effector protein was localized to mitochondria raised the possibility that Map harbors another motif that functions in mitochondria in evoking the signaling effect. Previous studies have implicated amino acids 101 to 152 of Map (also called the mitochondrial toxicity region [MTR] [4]) in altering mitochondrial morphology (18). To explore the significance of this motif, an EPEC-*map*+Map_Δ101–152_ mutant strain was generated (Tables S1 and S3; Fig. 6A). Map_Δ101–152_ was effectively translocated into the infected cells (Fig. 6B) and localized to mitochondria (Fig. S1I). Cell infection with EPEC-*map*+Map_Δ101–152_ resulted in a significant reduction in pMEK (data not shown), pERK (Fig. 6D), pEGFR (Fig. 6E), and ADAM10 activity (Fig. 6F), reaching the minimal values exhibited upon infection with EPEC-*map*. These data suggest that a Map MTR motif may be involved in promoting the ADAM10-MAPK signaling pathway.

10.1128/mBio.01397-20.9 TABLE S3 Plasmids. Download Table S3, DOCX file, 0.02 MB. Copyright © 2020 Ramachandran et al. 2020 Ramachandran et al. This content is distributed under the terms of the Creative Commons Attribution 4.0 International license.

### Map promotes Ca^2+^ mobilization from mitochondria.

**FIG 7:**
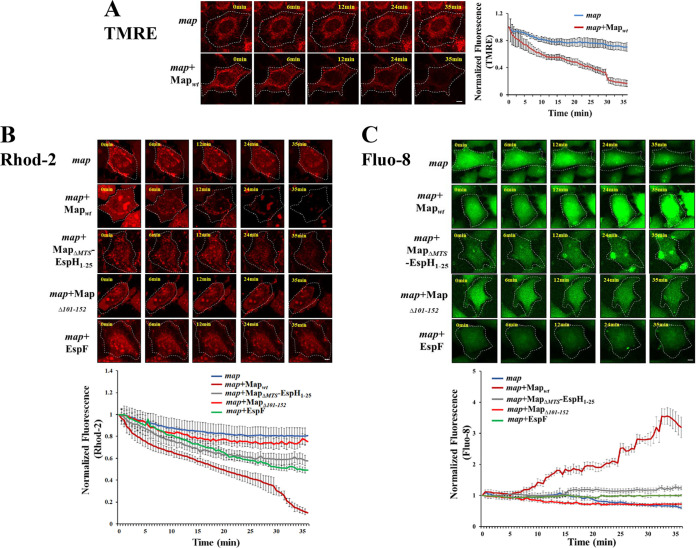
Map and EspF effects on mitochondrial membrane potential disruption and Ca^2+^ release into the cytoplasm. HeLa cells were labeled with the mitochondrial membrane potential indicator TMRE (A), the mitochondrial Ca^2+^ reporter Rhod-2 (B), or the fluorescent cytoplasmic Ca^2+^ label Fluo-8 (C), as described in Materials and Methods. Cells were then washed, infected with the indicated EPEC strains, and subjected to time-lapse confocal imaging (Movies S1 to S3). Representative images are shown. The fluorescence level of each marker was quantified at each time point and normalized to the fluorescence levels determined before bacterial exposure (0 min). Results are means ± SE for 30 cells recorded in 3 independent experiments. Bar = 2 μm.

Studies have shown that Map can disrupt the mitochondrial membrane potential (18, 29). We confirmed this in our experimental system by showing that cell infection with EPEC-*map*+Map_wt_ resulted in a continuous reduction in the fluorescence of the mitochondrial membrane potential reporter tetramethylrhodamine ethyl ester (TMRE). Only a moderate reduction in the TMRE fluorescent signal was observed in response to EPEC-*map* infection (Fig. 7A; Movie S1). The disruption of mitochondrial membrane potential can lead to Ca^2+^ efflux from mitochondria (reviewed in reference 30). Using the mitochondrial Ca^2+^-sensitive fluorescent reporter Rhod-2, we showed that cell infection with EPEC-*map*+Map_wt_ results in a sharp loss of mitochondrial Ca^2+^ levels, while infection with EPEC-*map*+Map_Δ101–152_ led to a slight reduction in mitochondrial Ca^2+^ compared to EPEC-*map*. Cell infection with EPEC-*map*+Map_ΔMTS_-EspH_1–25_ resulted in an intermediate effect (Fig. 7B; Movie S2). The efflux of mitochondrial Ca^2+^ may cause a parallel increase in cytoplasmic Ca^2+^. Indeed, cytosolic Ca^2+^ imaging by the Fluo-8 probe showed a parallel increase in cytoplasmic Ca^2+^ levels that was essentially spread throughout the cytoplasm in response to EPEC-*map*+Map_wt_ but not to EPEC-*map* or EPEC-*map*+Map_Δ101–152_ infection. Infection with EPEC-*map*+Map_ΔMTS_-EspH_1–25_ resulted in a slight increase in cytoplasmic Ca^2+^ (Fig. 7C; Movie S3). Taken together, these data suggest that mitochondrially targeted Map disrupts the mitochondrial membrane potential, leading to Ca^2+^ efflux from the mitochondria and Ca^2+^ influx into the infected cell cytoplasm. The MTR motif of MAP seems to play a major role in this effect.

10.1128/mBio.01397-20.2 MOVIE S1 Time-lapse confocal imaging of HeLa cells labeled with the mitochondrial membrane potential indicator, TMRE, and exposed to EPEC-*map* and EPEC-*map*+Map_wt_, infection. While mitochondrial membrane potential values changed only slightly over time in EPEC-*map*-infected cells, the membrane potential levels decreased dramatically over time in EPEC-*map*+Map_wt_-infected cells. Representative movies are shown, and quantitative analysis of the data is shown in Fig. 7A. Cells were imaged every 30 s in a 0- to ∼45-min time frame, and images of every minute are shown. Download Movie S1, AVI file, 4.7 MB. Copyright © 2020 Ramachandran et al. 2020 Ramachandran et al. This content is distributed under the terms of the Creative Commons Attribution 4.0 International license.

10.1128/mBio.01397-20.3 MOVIE S2 Time-lapse confocal imaging of HeLa cells labeled with the mitochondrial calcium indicator Rhod-2 AM and exposed to EPEC-*map*, EPEC-map+Map_wt_, EPEC-*map*+Map_ΔMTS_-EspH_1–25_, EPEC-*map*+Map_Δ101–152_, and EPEC-*map*+EspF infection. The mitochondrial calcium levels appeared to be unaltered in EPEC-*map*- and EPEC-*map*+Map_Δ101–152_-infected cells, whereas the mitochondrial calcium was only slightly altered over time in EPEC-*map*+Map_ΔMTS_-EspH_1–25_- and EPEC-*map*+EspF-infected cells. In contrast, the mitochondrial calcium levels diminished completely over time in EPEC-map+Map_wt_-infected cells. Notably, as time progressed in the latter case, the distribution of Rhod-2 fluorescence shifted from mitochondria to the nuclei of the infected cells. Representative movies are shown, and quantitative analysis of data is shown in Fig. 7B. Cells were imaged every 30 s in a 0- to ∼45-min time frame, and images of every minute are shown. Download Movie S2, AVI file, 15.9 MB. Copyright © 2020 Ramachandran et al. 2020 Ramachandran et al. This content is distributed under the terms of the Creative Commons Attribution 4.0 International license.

10.1128/mBio.01397-20.4 MOVIE S3 Time-lapse confocal imaging of HeLa cells labeled with the cytoplasmic calcium indicator, Fluo-8 AM, and exposed to EPEC-*map*, EPEC-*map*+Map_wt_, EPEC-*map*+Map_ΔMTS_-EspH_1–25_, EPEC-*map*+Map_Δ101–152_, and EPEC-*map*+EspF infection. The cytoplasmic calcium levels in the cells appeared to be transiently increasing (“blinking”) over time in EPEC-*map*- and EPEC-*map*+Map_Δ101–152_-infected cells. However, the overall average fluorescence level in the cell population did not significantly change over time. In EPEC-*map*+Map_ΔMTS_-EspH_1–25_- and EPEC-*map*+EspF-infected cells, there was a slight increase in cytoplasmic calcium. In contrast, in cells infected with EPEC-*map*+Map_wt_, the level of cytoplasmic calcium significantly increased over time. Representative movies are shown, and quantitative analysis of data is shown in Fig. 7C. Cells were imaged every 30 s in a 0- to ∼45-min time frame, and images of every minute are shown. Download Movie S3, AVI file, 15.1 MB. Copyright © 2020 Ramachandran et al. 2020 Ramachandran et al. This content is distributed under the terms of the Creative Commons Attribution 4.0 International license.

### Mitochondrial membrane potential disruption by CCCP induces Ca^2+^ extrusion and ADAM10/pERK activation.

**FIG 8:**
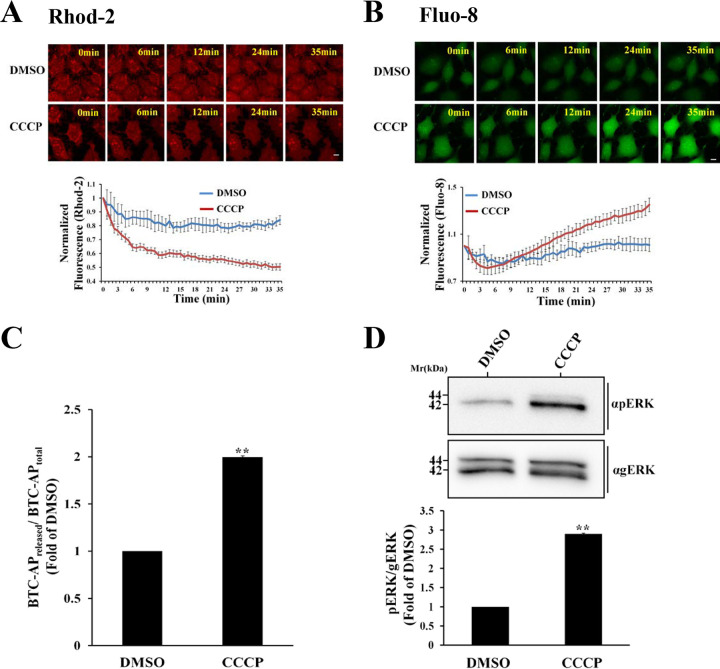
Disruption of mitochondrial membrane potential by the protonophore CCCP causes mitochondrial Ca^2+^ extrusion and an increase in cytoplasmic Ca^2+^ levels leading to ADAM10 and pERK activation. (A and B) Monitoring mitochondrial and cytoplasmic Ca^2+^ levels. HeLa cells were first labeled with the Rhod-2 and Fluo-8 fluorescent Ca^2+^ reporters, as described in Materials and Methods. Cells were then exposed to DMSO or the mitochondrial uncoupler CCCP and immediately subjected to time-lapse confocal imaging (Movies S4 and S5), as described in Materials and Methods. Representative images are shown in the upper panels and quantified fluorescence levels in the lower panels. The fluorescence level of each marker was quantified at each time point and normalized to the fluorescence levels determined before the addition of DMSO or CCCP. Results are means ± SE for 10 cells analyzed in 3 independent experiments. Bar = 2 μm. (C and D) Effects of CCCP on host ADAM10 sheddase activity and pERK levels. HeLa cells were treated with either DMSO or CCCP and then subjected to the ADAM10 sheddase assay or pERK/gERK analyses. Results are means and SE from 3 independent experiments. **, *P* < 0.005.

Previous studies suggest that Ca^2+^ can trigger ADAM10 (31–35) as well as MEK, ERK, and p38 activity (36–38). Based on these studies, we hypothesized that enrichment of the host cytoplasm with Ca^2+^ might prompt ADAM10 and MAPK activation. To tackle this hypothesis, HeLa cells were treated with the protonophore carbonyl cyanide *m*-chlorophenylhydrazone (CCCP), which results in the dissipation of the mitochondrial membrane potential, allowing the mitochondria to release accumulated Ca^2+^ and preventing its further accumulation in this organelle (39). Our results show that CCCP treatment resulted in a significant loss of mitochondrial Ca^2+^ (Fig. 8A; Movie S4), and a parallel increase in cytosolic Ca^2+^ (Fig. 8B; Movie S5). The activity of ADAM10 (Fig. 8C) and the levels of pERK (Fig. 8D) significantly increased in response to HeLa cell treatment with CCCP. These data, combined with data presented in Fig. 5 and 6, support the hypothesis that ADAM10 and the downstream ERK cascade are activated by Map-mediated Ca^2+^ extrusion from mitochondria and the consequent Ca^2+^ increase in the host cell cytoplasm.

10.1128/mBio.01397-20.5 MOVIE S4 Time-lapse confocal imaging of HeLa cells labeled with Rhod-2 AM and then exposed to DMSO or CCCP. While the level of mitochondrial calcium in the cells appears to be only slightly reduced over time in DMSO-treated cells, mitochondrial calcium levels were rapidly and significantly diminished upon CCCP treatment. A representative movie is shown, and the quantitative data analysis is shown in Fig. 8A. Cells were imaged every 30 s in a 0- to ∼45-min time frame, and images of every minute are shown. Download Movie S4, AVI file, 4.7 MB. Copyright © 2020 Ramachandran et al. 2020 Ramachandran et al. This content is distributed under the terms of the Creative Commons Attribution 4.0 International license.

10.1128/mBio.01397-20.6 MOVIE S5 Time-lapse confocal imaging of HeLa cells labeled with Fluo-8 AM and exposed to DMSO or CCCP. The level of cytoplasmic calcium increased slightly over time in the DMSO-treated cells. In CCCP-treated cells, the cytoplasmic calcium levels increased significantly over time. A representative movie is shown, and the quantitative data analysis is shown in Fig. 8B. Cells were imaged every 30 s in a 0- to ∼45-min time frame, and images of every minute are shown. Download Movie S5, AVI file, 3.5 MB. Copyright © 2020 Ramachandran et al. 2020 Ramachandran et al. This content is distributed under the terms of the Creative Commons Attribution 4.0 International license.

### Map induces apoptosis.

**FIG 9:**
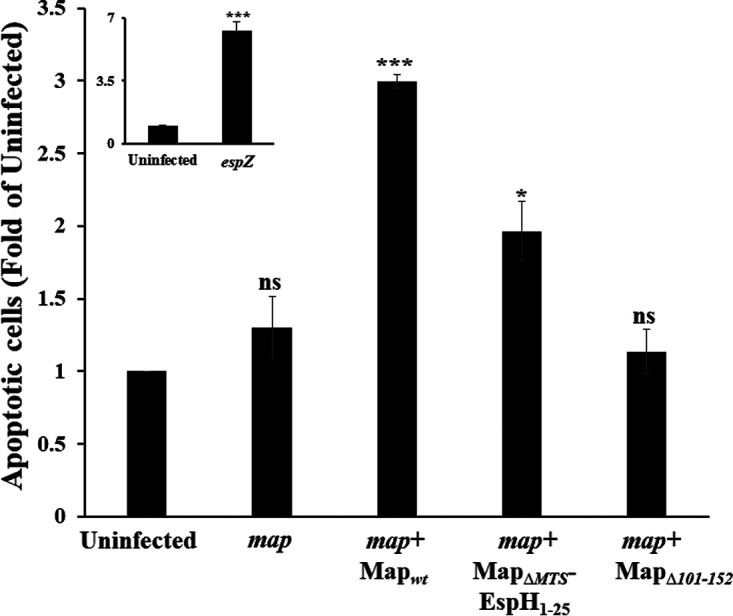
Map induces host cell apoptosis. HeLa cells infected with the indicated EPEC strains were subjected to a flow cytometry-based apoptosis assay, as described in Materials and Methods. The fraction of EPEC-exposed apoptotic cells (i.e., annexin V-positive and PI-positive cells) was normalized to the fraction of apoptotic cells detected in uninfected cells. The fractions of these cells in uninfected cells and in EPEC-*map*-, EPEC-*map*+Map_wt_-, EPEC-*map*+Map_ΔMTS_-EspH_1–25_-, and EPEC-*map*+Map_Δ101–152_-infected cells were 8%, 24%, 11%, 16%, and 10%, respectively. The fraction of apoptotic cells in EPEC-*espZ*-infected cells, which was greater than 50% and ∼6-fold higher than the fraction of apoptotic cells in uninfected cells (inset), served as a positive control for these experiments, as previous studies have shown that an EPEC-*espZ* mutant increases dramatically the levels of apoptotic and necrotic cells (70). The fractions of cells that were dually labeled with annexin V and PI were negligible (≤6%) in all experiments. Results are means and SE from 3 independent experiments. ***, *P* < 0.0005; *, *P* < 0.05; ns, nonsignificant (*P* > 0.05).

Activation of the ERK cascade is linked to cell survival and death (40, 41). Mitochondrial dysfunction, membrane potential perturbation, fragmentation (fission), and redistribution have all been shown to trigger cell death (30). Data have also linked Ca^2+^ signaling, mitochondria, and various forms of cell death (42, 43). Translocated Map seems to impact Ca^2+^ compartmentalization (Fig. 7B and C), and active ERK (Fig. 6D), EGFR (Fig. 6E), and ADAM10 (Fig. 6F) levels in a mitochondrial targeting-dependent manner. We, therefore, postulated that these Map-mitochondria dependent processes could promote host cell death. Data in Fig. 9 unambiguously show that cell infection with EPEC-*map*+Map_wt_ resulted in a significant increase in the appearance of apoptotic cells compared to cells infected with EPEC-*map* or uninfected cells. This phenomenon was dependent on the ability of Map to target mitochondria, as apoptotic-cell levels in EPEC-*map*+Map_ΔMTS_-EspH_1–25_- and EPEC-*map*+Map_Δ101–152_-infected cells were significantly reduced compared to those in EPEC-Map_wt_-infected cells.

## DISCUSSION

Map was shown to target host cell mitochondria, *in vitro* and *in vivo* (17, 18, 29). Map has also been suggested to play a significant role in bacterial colonization and the induction of the diarrheal effect (21, 29, 44, 45). Despite its importance as a virulent factor and the fact that most Map localizes at steady state in mitochondria, the functional significance of Map-mitochondrion interactions is not known. Here, we demonstrate, for the first time, that early in infection, mitochondrial targeting of Map, particularly via its MTR motif, plays a role in activating the sheddase activity of ADAM10 (Fig. 5F and 7E). This activity may, in turn, induce the pEGFR (Fig. 3E, 5C, and 6E) and possibly another EGFR family member, ErbB4, because BTC has been shown to activate both the EGFR and ErbB4 (46). The activation of the EGFR may then elicit the MEK/ERK/MAPK signaling cascade (Fig. 3A, 5B, and 6D). Our data also suggest that Map can activate p38 (Fig. 3F). Previous studies suggested that EPEC and similarly, enterohemorrhagic E. coli (47) rapidly induce T3SS-dependent ERK, p38, and JNK in infected cells (15, 16). Here, we propose that Map is the effector that activates ERK and p38. All in all, these effects of Map do not depend on the ability of the effector to activate Cdc42 and induce actin-rich filopodia, because the mutant Map_WxxxA_, which fails to induce Cdc42 (Fig. S1E), retains the capacity to induce the ADAM10-MAPK signaling (Fig. 5). Thus, early upon translocation, Map localizes to infection sites, but not to mitochondria, where it exerts Rho GEF signaling to remodel the host actin cytoskeleton. In parallel, the effector fraction targeting mitochondria evokes the ADAM10-MAPK signaling, independently.

**FIG 10:**
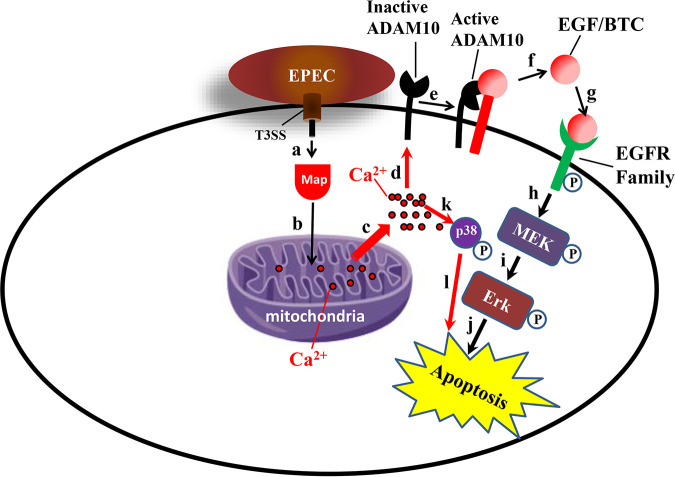
Schematic model of EPEC-induced ADAM10-MPAK signaling by Map. Upon translocation into the host cell (a), Map targets mitochondria (b), where it disrupts the mitochondrial membrane potential, resulting in the release of mitochondrial Ca^2+^ into the host cytoplasm (c). Elevated cytoplasmic Ca^2+^ levels then stimulate the ADAM10 sheddase activity (d), which results in the release of EGF/BTC (e). The released ligands (f) bind EGFR (g), which in turn activates (phosphorylates) the EGFR-MEK-ERK signaling cascade (h and i). This sequence of events can lead to host cell apoptosis (j). The rise in cytoplasmic Ca^2+^ may also directly trigger the activity of the MAPK p38 (k), which can also evoke apoptosis (l).

We also show that Map targeting of mitochondria mediates the disruption of the mitochondrial membrane potential and Ca^2+^ release from mitochondria into the host cytoplasm (Fig. 7). Evidence suggests that the activity of ADAM10, MEK, ERK, and p38 is regulated by Ca^2+^ (31–33, 37, 48, 49). Thus, a Map-mediated increase in cytoplasmic Ca^2+^ could have contributed to the activation of these enzymes. This hypothesis may also be supported by the observation that disruption of mitochondrial membrane potential by the protonophore CCCP increases cytoplasmic Ca^2+^ levels and the ADAM10 and ERK activities (Fig. 8). A proposed model for the action of Map is presented in Fig. 10.

How does the activation of ADAM10-ERK and p38 relate to EPEC pathogenesis? MAPKs can act as a double-edged sword in promoting cell death and survival (50). In most cases, ERK signaling promotes cell survival, e.g., by activating prosurvival BCL2 proteins and repressing prodeath proteins (BAD, BIM, BMF, and PUMA). However, active ERK, under certain conditions, such as nutrient deprivation, can also drive the expression of the prodeath proteins to control autophagy or apoptosis (reviewed in references 51 and 52). Similarly, previous studies suggested that activation of p38 is required for inducing the mitochondrial death pathway (53–55). Hence, it is possible that by targeting mitochondria, Map induces host cell deprivation, which, combined with ERK and p38 activation, triggers host cell apoptosis (Fig. 9).

Similar to Map, the type III secreted effector EspF targets host mitochondria via an N-terminal targeting signal to cause mitochondrial dysfunction and cell death (56–58). However, in our screen, EspF was not identified as an effector that stimulates pERK (Fig. 2A). This concern was investigated by generating new EPEC strains in which EspF was expressed in the background of EPEC-*map* (EPEC-*map*+EspF) or EPEC-*map*,*espF* (EPEC-*map*,*espF*+EspF) (Table S1). We used these bacterial strains to infect HeLa cells under the experimental conditions used to investigate the role of Map. The levels of translocated EspF upon cell infection were nearly identical (Fig. S1J). Infection with either EPEC strain resulted in a minor increase in host pERK levels compared to EPEC-*map*+Map_wt_ (Fig. S1K and L). Infection with EPEC-*map*+EspF also caused a slight decrease in mitochondrial Ca^2+^ levels (Fig. 7B; Movie S2) and a corresponding increase in cytoplasmic Ca^2+^ levels (Fig. 7C; Movie S3). Confocal imaging revealed that the localization of translocated EspF was confined to host cell areas juxtaposed to the infection sites, where the effector protein only partially colocalized with the mitochondrial marker Hsp60 (Fig. S1M). These observations, which are consistent with our most recent discoveries (59), suggest that early upon infection with EPEC-*espF*, Map is translocated and efficiently targets mitochondria, where it initiates the cascade of events that leads to pERK activation. In contrast, infection with EPEC-*map* allows EspF translocation, which only partially targets mitochondria and thereby exhibits a limited capacity to induce pERK. Additionally, Map’s MTR motif, which we show here to be critical for promoting ADAM10-MAPK signaling, does not exist in EspF (18). Nevertheless, at a later infection time, EspF may reach the host mitochondria more effectively to promote cell death that is superimposed on the process that has been started by Map.

By targeting mitochondria, Map induces an apoptotic effect early upon infection. However, EspH may counteract this effect at a later infection time (13). These opposing effects may delay the enforcement of the host cell death and therefore be required for establishing bacterial colonization and successful infection. The early Map-dependent effects could be physiologically important, because *in vivo* infection studies suggested that Map expression is suppressed at an early colonization phase, and when Map expression is reduced, EPEC colonization is also significantly reduced (44). Thus, the downregulation of Map could be another mechanism that terminates its effects. Many intestinal microbial pathogens modulate mitochondrial cell death to achieve successful infection (60). Hence, mitochondrial targeting, e.g., by Map, may represent a universal event that plays a role in establishing bacterial pathogens’ colonization of the gut. The mechanisms underlying these effects are presently unknown. One intriguing hypothesis is that by causing mitochondrial dysfunction, pathogens build a niche where they succeed in competing with nearby commensal bacteria (microbiota) whose viability depends upon functional mitochondria of the host (61).

The precise mechanism by which Map evokes ADAM10 activation through mitochondrial targeting is still not well understood. Recent studies have shown that membrane externalized phosphatidylserine is required for activating the ADAM10 sheddase activity (62). Hence, an interesting hypothesis would be that the induction of mitochondrial cell death by Map is caused by phosphatidylserine externalization. This process is shown here to take place because the detection of cell apoptosis utilizes the annexin V reporter, which binds specifically to externalized phosphatidylserine (Fig. 9). Finally, most recent findings may signify the importance of ERK in EPEC infection by showing that the effector NleD selectively cleaves several MAPKs but not ERK (63), probably leaving it intact for activation by Map.

## MATERIALS AND METHODS

### Bacterial strains, antibodies, primers, plasmids, and site-directed mutagenesis.

Bacterial strains, antibodies, plasmids, and primers used in this study are listed in Tables S1 to S4, respectively. For constructing the EPEC-*espH*,*map* double mutant strain (Table S1), λ Red recombinase system (64) was used to mutate *map* in EPEC-*espH*::Kan strains. The upstream and downstream recombination sequences were PCR amplified from the genomic DNA of EPEC-wt using primer pairs 1371-4197 and 1495-4198, respectively (Table S4). Primers 1354 and 1355 (Table S4) were used to amplify a chloramphenicol cassette from pKD3 (Table S3). DNA of the *map*::Cam allele was made by isothermal assembly of these three PCR fragments (65), followed by electroporation into the EPEC *espH*::Kan strain containing pKD46 (Table S3) containing λ Red genes (γ, β, and exo). The desired mutants were selected, and pKD46 was cured at 42°C. The mutation was verified using sequencing and PCR with flanking primers. The Map mutants Map_ΔMTS_ and Map_WxxxA_, which were subcloned into the pSA10 plasmid, were constructed using the 5′-phosphorylated primer pairs MTS delta F-MTS delta R and WxxxA F-WxxxA R, respectively (Table S4). Mutations were introduced by a site-directed mutagenesis method using Platinum Superfi II DNA polymerase, based on protocol B for deletions in the manufacturer’s protocol (https://assets.thermofisher.com/TFS-Assets/LSG/manuals/MAN0014883_Platinum_SuperFi_PCR_MM_UG.pdf) (Thermo Fisher). The Map_ΔTRL_, Map_ΔMTS_-EspH_1–25_, and Map_Δ101–152_ mutants were prepared by the standard Gibson assembly method (65), using the Gibson assembly master mix (number E2611; NEB) and the respective linearization primers and the gblock (Table S4). The wild-type and mutated Map constructs were electroporated into EPEC-*map*, and Amp^r^ EPEC-*map*+Map_wt_ strains were isolated (Table S1). The Map_wt_-eGFP plasmid (Table S3) was constructed by the Gibson assembly method using mCherry-Map_wt_ (Table S3) as the template. The primers EGFP F, EGFP R, Map EGFP F, and Map EGFP R (Table S4) were used for generating the Map-eGFP plasmid. All mutations were confirmed by sequencing. All bacterial strains used in this study were grown in Luria-Bertani medium supplemented with appropriate antibiotics (Table S1).

10.1128/mBio.01397-20.10 TABLE S4 List of primers and their usage. Download Table S4, DOCX file, 0.01 MB. Copyright © 2020 Ramachandran et al. 2020 Ramachandran et al. This content is distributed under the terms of the Creative Commons Attribution 4.0 International license.

### Cell culture, ectopic expression of Map, and ADAM10 silencing by siRNA.

HeLa and CaCo-2BBe Tet-off cells were cultured as described elsewhere (13). Plasmid DNAs (eGFP, Map-eGFP, MTS-eGFP, mCherry, and mCherry-Map) (Table S3) were transiently transfected into HeLa cells (∼60% confluence) for 15 h at 37°C, using the TransIT-X2 transfection reagent protocol (MIR 6004; Mirus, Madison, WI). For silencing ADAM10 expression in HeLa cells (∼40% confluence), the TransIT-X2 transfection reagent was used to transfect the ADAM10 (50 nM; no. M-002000-00-0005; Dharmacon) or scrambled siRNA (50 nM; no. D-001206-14-05; Dharmacon) for 72 h at 37°C. ADAM10 expression was validated by Western blotting using specific antibodies (Table S2).

### Bacterial growth, activation, and infection.

The T3SS of all bacterial strains used in the study was activated (3 h at 37°C and 5% CO_2_) before infection, as described elsewhere (13). In EPEC-*map*+Map-infected cells, Map expression was induced by supplementing the activation medium with 0.2 mM isopropyl-β-d-thiogalactopyranoside (IPTG; Promega, Madison, WI) during the last 30 min of activation. All infections were performed in a CO_2_ incubator (37°C, 5% CO_2_, 90% humidity) for 30 min, as described elsewhere (13).

### Confocal microscopy. (i) Immunofluorescence labeling of fixed cells and confocal imaging.

The procedures employed for confocal microscopy were described previously (13). Briefly, after infection, cells were fixed with 4% paraformaldehyde, F-actin was labeled with Texas Red (TR)-phalloidin, and the bacterial and HeLa cell DNA was labeled with DAPI (4′,6-diamidino-2-phenylindole). Primary and fluorescence-labeled secondary antibodies used in this study are listed in Table S2. Cells were mounted and visualized using an Olympus FV-1200 laser scanning confocal microscope equipped with a 60×, 1.42-numerical-aperture oil immersion objective. The excitation wavelengths were 405 nm, 488 nm, 561 nm, and 633 nm, and the emission filter passbands were 430 to 470 nm, 505 to 525 nm, 575 to 625 nm, and 650 to 720 nm for DAPI, green, red, and far-red fluorescence, respectively. Confocal sections were acquired at *z*-axis intervals of 0.5 or 1 μm. The levels of F-actin at the infection sites were analyzed using Fiji (NIH) (66). A maximum-intensity projection was generated for each stack. The regions of cell-associated EPEC microcolonies were manually defined using the polygon selections tool of Fiji and were termed infection sites. The average pixel intensity (PI) at the infection site (PI_i_) was measured and expressed as normalized fluorescence from the average PI of an identically defined uninfected area located near the infecting microcolony (PI_u_), as follows: normalized fluorescence = (PI_i_ − PI_u_)/PI_u_. For colocalization analyses, line intensity profiles of the anti-hemagglutinin (HA) (Map) and anti-Hsp60 (mitochondria) fluorescence were generated, as previously described (13), and are depicted in Fig. S1I. Basically, a line was drawn across a randomly chosen confocal section showing Map and mitochondrial staining, and the fluorescence intensity along the line of the two different channels was generated using the intensity profile tool (plot-profile plug-in) of Fiji (NIH). Colocalized labeling was scored when the fluorescence intensity copeaked at a given place along the line (indicated by the green arrows in the intensity profile plots in Fig. S1I). Data are presented as percentages of colocalizing and noncolocalizing fluorescence intensity peaks determined in 10 such intensity profile determinations.

### (ii) Live-cell imaging.

Cells were seeded on 8-well ibiTreat microslides (catalog no. 80826; ibidi) for 2 days (∼70% confluence). Cells were washed twice with plain Dulbecco modified Eagle medium (DMEM) and then incubated with either the fluorescent mitochondrial membrane potential indicator tetramethylrhodamine ethyl ester (TMRE; 20 nM) (T669; Thermo Fisher), the fluorescent mitochondrial Ca^2+^ indicator Rhod-2 AM (1 μM; ab142780; Abcam), or the fluorescent cytoplasmic Ca^2+^ indicator Fluo-8 AM (1 μM; ab142773; Abcam) in DMEM for 15 min at 37°C in the CO_2_ incubator. Cells were then washed three times with Hanks’ balanced salt solution (HBSS) (no. 02-016-1A; Biological Industries) and exposed to EPEC strains or the protonophore carbonyl cyanide *m*-chlorophenyl hydrazone (CCCP; 20 μM) (ab141229; Abcam) for 30 min at 37°C in a CO_2_ incubator. Cells were then immediately subjected to time-lapse confocal imaging using an Olympus FV-1200 laser scanning confocal microscope (Olympus, Japan) equipped with a temperature and CO_2_ incubator, using a 40×, 0.95-numerical-aperture air objective. The excitation wavelengths were 488 nm or 561 nm and the emission filter passbands were 505 to 525 nm and 575 to 625 nm for green and red fluorescence, respectively. Scans were taken every 30 s for 35 min under identical conditions. Fluorescence intensity at all time points was measured using the polygonal selection tool of Fiji (NIH) to select the desired region of interest (ROI). In the case of TMRE and Rhod-2 AM, the ROI was confined to the labeled mitochondria. In the case of Fluo-8 AM, the ROI included the entire cell. Data are presented as the average fluorescence intensity of 10 cells normalized to their fluorescence intensity at time zero. In the supplemental movies, time and titles were introduced using the time stamper and label tool of Fiji (NIH).

### SDS-PAGE and Western blotting.

Cells (∼70% confluence) were infected with the respective bacteria, as described above. Cells were then washed with phosphate-buffered saline (PBS) three times, and equal numbers of cells were lysed in sample buffer (40% glycerol, 12% SDS, 0.2 M Tris-HCl [pH 6.8], and 100 mM dithiothreitol) supplemented with bromophenol blue, followed by heating (95°C for 5 min) and vortex shaking (10 min). Proteins separated by SDS-PAGE (Bio-Rad Mini-Protean Tetra system; 40 mA, 30 min) were transferred to a nitrocellulose membrane (Bio-Rad Trans-Blot Turbo; 2.5 A, 10 min). Membranes were then blocked (2.5% [wt/vol] bovine serum albumin [BSA] plus 1% [wt/vol] skim milk) for 1 h at 22°C with agitation, washed with TBST (20 mM Tris [pH 8.0], 150 mM NaCl, and 0.1% Tween 20), and probed with the appropriate anti-phosphorylated-EGFR (pEGFR) or MAPK antibodies (Table S2). For stripping, the membranes were incubated in stripping buffer (62.5 mM Tris-HCl [pH 6.8], 100 mM 2-mercaptoethanol, 2% SDS) for 20 min at 55°C. Membranes were then washed with TBST and reprobed with anti-general-EGFR (gEGFR) or MAPK antibodies (Table S2). A Fusion FX Spectra imager (Vilber Smart Imaging, Collègien, France) was used to image the membranes. Band intensity was measured by Fiji (NIH). The band intensity of the phosphorylated protein was normalized to the band intensity of the total protein. The value obtained was further normalized to the protein level of the control experiment.

### GTPase activity assay.

The Rac/Cdc42 (p21) binding domain (PBD) of the human p21 activated kinase 1 (PAK1) protein fused to GST (pGEXTK-Pak1 70–117 [no. 12217; Addgene]) was expressed and coupled to glutathione Sepharose 4b beads (GST-PBD beads), as described (67). HeLa cells (70% confluence) were infected with various EPEC strains for 30 min at 37°C, then washed with cold PBS, and lysed in ice-cold lysis buffer (50 mM Tris [pH 7.6], 150 mM NaCl, 1% Triton X-100, 20 mM MgCl_2_) supplemented with protease inhibitors (phenylmethylsulfonyl fluoride [PMSF], leupeptin, and aprotinin). Lysates were sonicated for 10 s and centrifuged (14,000 rpm, 10 min, 4°C). The protein concentration of the lysates was determined by the bicinchoninic acid (BCA) protein assay method (catalog no. 23227; Thermo Fisher Scientific). Lysates (1 mg/ml) were incubated with 200 μg of GST-PBD beads for 30 min at 4°C and mixed by end-over-end rotation. Beads were washed three times with lysis buffer and dried with a Hamilton syringe, and associated proteins were analyzed by SDS-PAGE followed by Western blotting, as before.

### Effector translocation assay.

The effector translocation assay was performed as described elsewhere (13). Briefly, HeLa cells (∼70% confluence) grown in 6-well plates were infected with preactivated EPEC strains for 30 min at 37°C. Cells were lysed with ice-cold lysis buffer (100 mM NaCl, 1 mM EDTA, 10 mM Tris-HCl [pH 7.4], 0.5% [vol/vol] NP-40) supplemented with protease and phosphatase inhibitors. Detergent-soluble (host cytoplasm and membranes containing the translocated protein effector) and -insoluble (bacteria and associated protein effectors) fractions were separated by centrifugation (16,000 × *g*, 4°C, 10 min) and analyzed for the presence of Map by Western blotting. The level of translocated effector was calculated by normalizing the levels of effector present in the NP-40-soluble fraction to the levels of the effector detected in the detergent-insoluble fraction.

### Sheddase activity assay.

The ectodomain shedding assay was performed essentially as described elsewhere (25). Briefly, HeLa cells grown on a 6-well plate (∼60% confluence) were transfected with betacellulin-alkaline phosphatase (BTC-AP) or with TGF-α–AP. After 24 h, cells were infected with the appropriate EPEC strains for 30 min at 37°C. Medium containing bacteria was aspirated and replaced with Opti-MEM (reduced serum medium; no. 31985-047; Gibco) for 1 h at 37°C. The medium was then collected, and the cells were lysed in lysis buffer (2.5% [vol/vol] Triton X-100 in triple-distilled water [TDW]). Cell lysates were centrifuged (13,000 rpm, 5 min), and the supernatants were diluted 1:10 in a Triton–double-distilled-water (DDW) solution. Samples (100 μl) derived from media and cell lysates were placed in a 96-well plate and incubated with 4-nitrophenyl phosphate (100 μl) for 45 min at 37°C in the CO_2_ incubator. The levels of TGF-α–AP and BTC-AP released to the medium and in the cell lysates were determined by absorbance at 405 nm, using a Synergy H1 microplate reader (Biotek). The levels of sheddase activity were expressed as AP activity levels measured in the supernatant (i.e., AP_released_) normalized to the total AP activity (i.e., AP_released_ + AP_lysate_). In experiments measuring sheddase activity in CCCP-treated cells, cells were first transfected with BTC-AP and then treated with CCCP (20 μM, 30 min, 37°C).

### Apoptosis assay.

Apoptosis assay was performed using the MEBCYTO apoptosis kit (annexin V-fluorescein isothiocyanate [FITC] kit; no. 4700; MBL) per user instructions. Briefly, HeLa cells were grown in a 6-well plate (∼70% confluence) and infected with the respective EPEC strains, as described in Materials and Methods. Cells were washed once with PBS, trypsinized, and washed once with serum-containing DMEM and once with PBS. They were suspended in 85 μl binding buffer, treated with annexin V-FITC and propidium iodide for 15 min at 22°C, and analyzed by flow cytometry (Cell Stream flow cytometer; Merck). The fractions of viable cells (annexin V negative, propidium iodide negative), early apoptotic cells (annexin V positive, propidium iodide negative), late apoptotic cells (annexin V positive, propidium iodide positive), and necrotic cells (annexin V negative, propidium iodide positive) were determined in each sample. Voltage settings for forward scatter (FSC) and side scatter (SSC) and fluorescence channels (488 and 561 nm) were kept constant for all samples. Data for 10,000 cells were recorded in each experiment.

### Statistical analyses.

Results are presented as means and standard errors (SE) of the means. A two-tailed Student's *t* test determined statistical significance. A *P* value of <0.05 indicates a statistically significant difference.
