# Supplementary material for: Mitochondrial Targeting of the Enteropathogenic Escherichia coli Map Triggers Calcium Mobilization, ADAM10-MAP Kinase Signaling, and Host Cell Apoptosis
Source: mBio. 2020 Sep 15;11(5):e01397-20. doi: 10.1128/mBio.01397-20 (PMC7492733; doi:10.1128/mBio.01397-20)
Supplement: TABLE S1 [file mBio.01397-20-st001.docx]

**Table S1: Bacterial EPEC strains (all mutant strains are derivatives of E2348/69)**

| **Strain Name** | **Description** | **Reference and Comments** |
| --- | --- | --- |
| E2348/69 (*wt*) | EPEC-*wt* isolate, serotype O127:H6, NA | J. Kaper, (1) |
| SN191 (*escV*) | *escV*::miniTn5,Kn | (2), EPEC mutated in the T3SS |
| ICC202 (*map*) | *map::Kn* | (3), LEE effector *map* mutant |
| XT111 (*espH*) | *espH::Kn*  EPEC-*espH* | (4), LEE effector *espH* mutant |
| UMD874 (*espF*) | *espF::Kn* | (5), LEE effector *espF* mutant |
| SE1207 (*espG1*,*espG2*) | *espG1,espG2::*Kn | (6), LEE effector *espG1* and non-LEE effector *espG2* mutant |
| EM3458  (*tir*) | *Tir::*Cm | (7), LEE effector; *tir* mutant |
| SK5566 | *espZ::Kn* | (8), LEE effector; *espZ* mutant |
| RP8153  (*espH,map*) | *espH*::Kn, map::cam | This study, LEE effectors *espH* and *map* mutants |
| BA1178 | *map*+Map*_wt_*, Amp | This study, *map* strain complemented with a pSA10-Map*_wt_* plasmid |
| BA1169 | *escV*+Map*_wt_*, Amp | This study, *escV* strain complemented with a pSA10-Map*_wt_* plasmid |
| BA1202 | *map*+Map*_WxxxA_*, Amp | This study, *map* strain complemented with a psA10-Map*_WxxxA_* encoding plasmid. Map-HA in which E78 has been substituted for A to generate an inactivated Rho GEF domain (WxxxA) (9) |
| BA1203 | *map*+Map_Δ_*_TRL_*, Amp | This study, *map* strain complemented with a psA10Map_Δ_*_TRL_* encoding plasmid. Map-HA in which the C-terminal TRL (201-203) PDZ type I binding motif was deleted in-frame (9) |
| BA1222 | *map*+Map*_∆MTS-_EspH_1-25_*, Amp | This study, *map* strain complemented with a psA10-Map*_∆MTS-_EspH*_1-25_ encoding plasmid. In this mutant, the N-terminal 2-25 amino acids of EspH was fused to the N-terminus of Map_∆_*_MTS_* (Map_∆_*_MTS_* is deleted of 1-44aa of Map) |
| BA1223 | *map*+Map_Δ_*_101-152_*, Amp | This study, *map* strain complemented with a psA10-Map_Δ_*_101-152_* encoding plasmid. In this mutant, the mitochondrial toxicity region (aa 101-152) of Map-HA has been deleted (9) |
| BA1211 | *map,espF*+EspF, Amp | This study, *map, espF* double mutant strain complemented with a C-terminally Flag-tagged EspF encoding plasmid |
| BA1212 | *map,espF*+Map*_wt_*, Amp | This study, *map, espF* double mutant strain complemented with a psA10-Map*_wt_* encoding plasmid |

References

1. Levine MM, Bergquist EJ, Nalin DR, Waterman DH, Hornick RB, Young CR, Sotman S. 1978. Escherichia coli strains that cause diarrhoea but do not produce heat-labile or heat-stable enterotoxins and are non-invasive. Lancet 1:1119-22.

2. Nadler C, Shifrin Y, Nov S, Kobi S, Rosenshine I. 2006. Characterization of enteropathogenic Escherichia coli mutants that fail to disrupt host cell spreading and attachment to substratum. Infect Immun 74:839-49.

3. Wong AR, Clements A, Raymond B, Crepin VF, Frankel G. 2012. The interplay between the Escherichia coli Rho guanine nucleotide exchange factor effectors and the mammalian RhoGEF inhibitor EspH. MBio 3:00250-11.

4. Tu X, Nisan I, Yona C, Hanski E, Rosenshine I. 2003. EspH, a new cytoskeleton-modulating effector of enterohaemorrhagic and enteropathogenic Escherichia coli. Mol Microbiol 47:595-606.

5. McNamara BP, Koutsouris A, O'Connell CB, Nougayrede JP, Donnenberg MS, Hecht G. 2001. Translocated EspF protein from enteropathogenic Escherichia coli disrupts host intestinal barrier function. J Clin Invest 107:621-9.

6. Elliott SJ, Krejany EO, Mellies JL, Robins-Browne RM, Sasakawa C, Kaper JB. 2001. EspG, a novel type III system-secreted protein from enteropathogenic Escherichia coli with similarities to VirA of Shigella flexneri. Infection and immunity 69:4027-33.

7. Mills E, Baruch K, Aviv G, Nitzan M, Rosenshine I. 2013. Dynamics of the type III secretion system activity of enteropathogenic Escherichia coli. mBio 4.

8. Berger CN, Crepin VF, Baruch K, Mousnier A, Rosenshine I, Frankel G. 2012. EspZ of enteropathogenic and enterohemorrhagic Escherichia coli regulates type III secretion system protein translocation. MBio 3:00317-12.

9. Dean P, Kenny B. 2009. The effector repertoire of enteropathogenic E. coli: ganging up on the host cell. Curr Opin Microbiol 12:101-9.
