# Supplementary figures and images for: Mitochondrial Targeting of the Enteropathogenic Escherichia coli Map Triggers Calcium Mobilization, ADAM10-MAP Kinase Signaling, and Host Cell Apoptosis
Source: mBio. 2020 Sep 15;11(5):e01397-20. doi: 10.1128/mBio.01397-20 (PMC7492733; doi:10.1128/mBio.01397-20)

**Fig. S1**

**A**

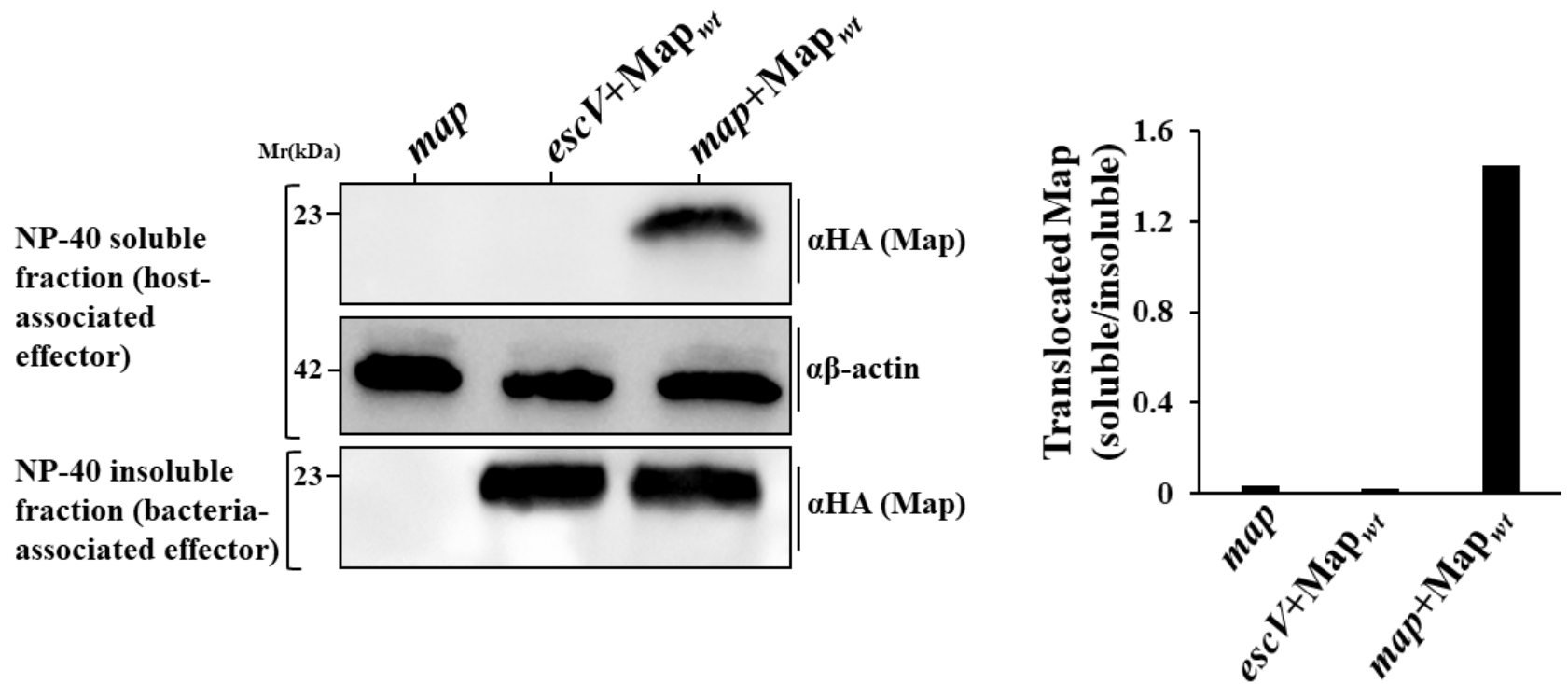

**Fig. S1**

**B**

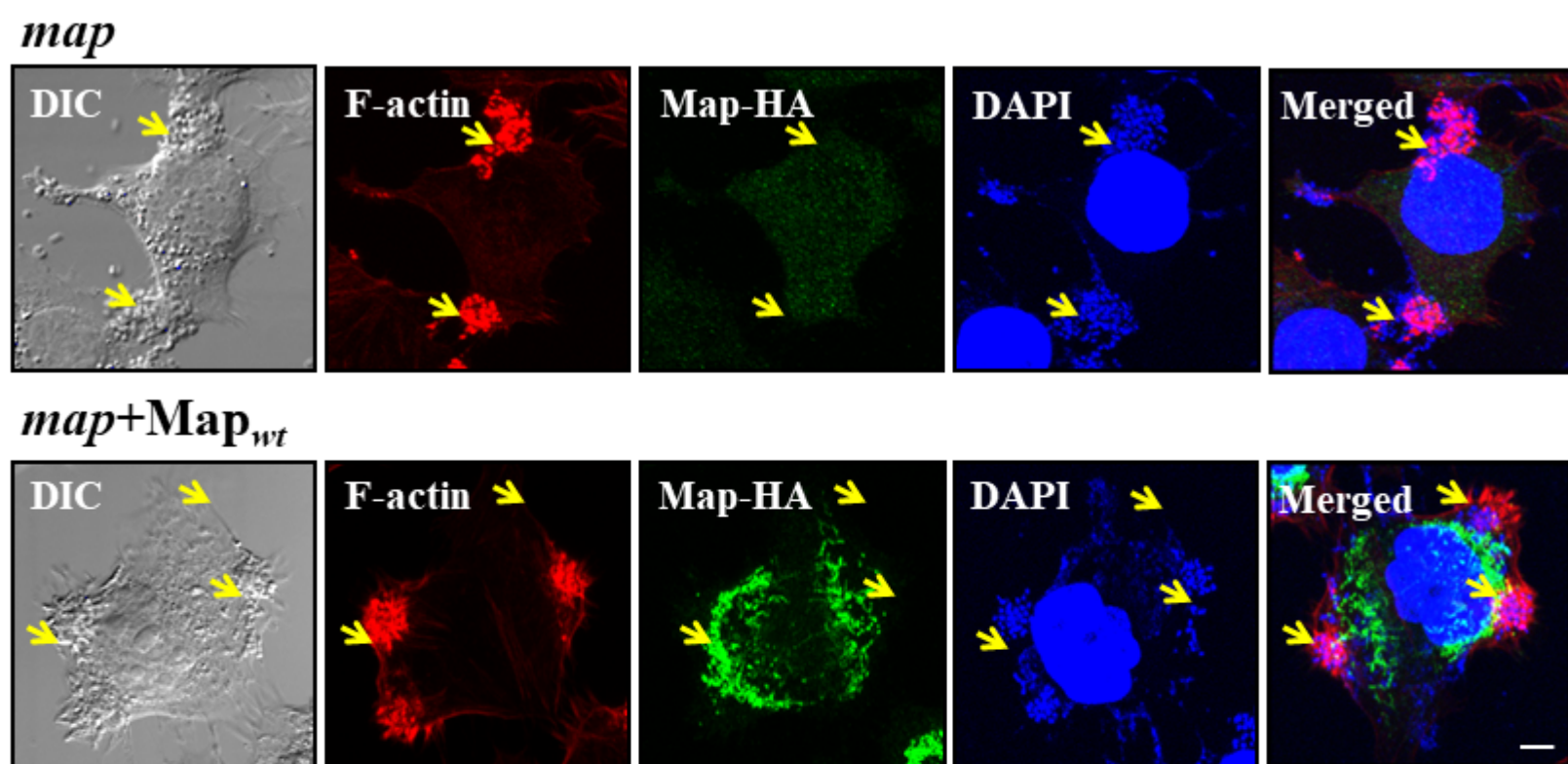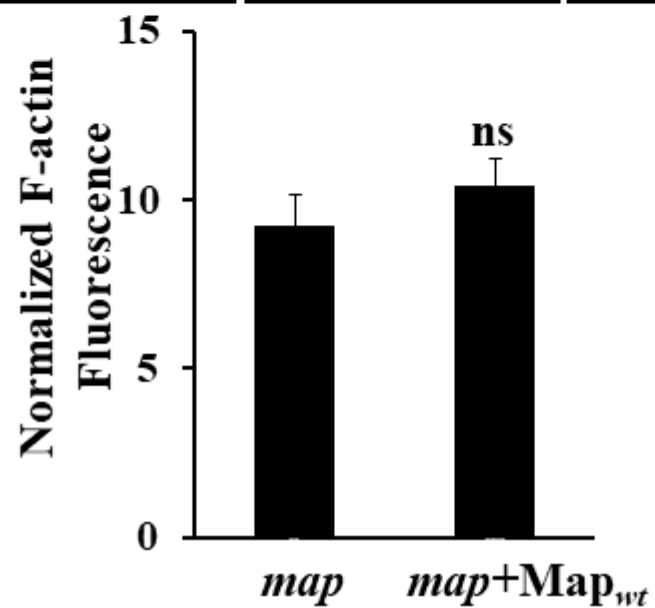

**Fig. S1**

**C**

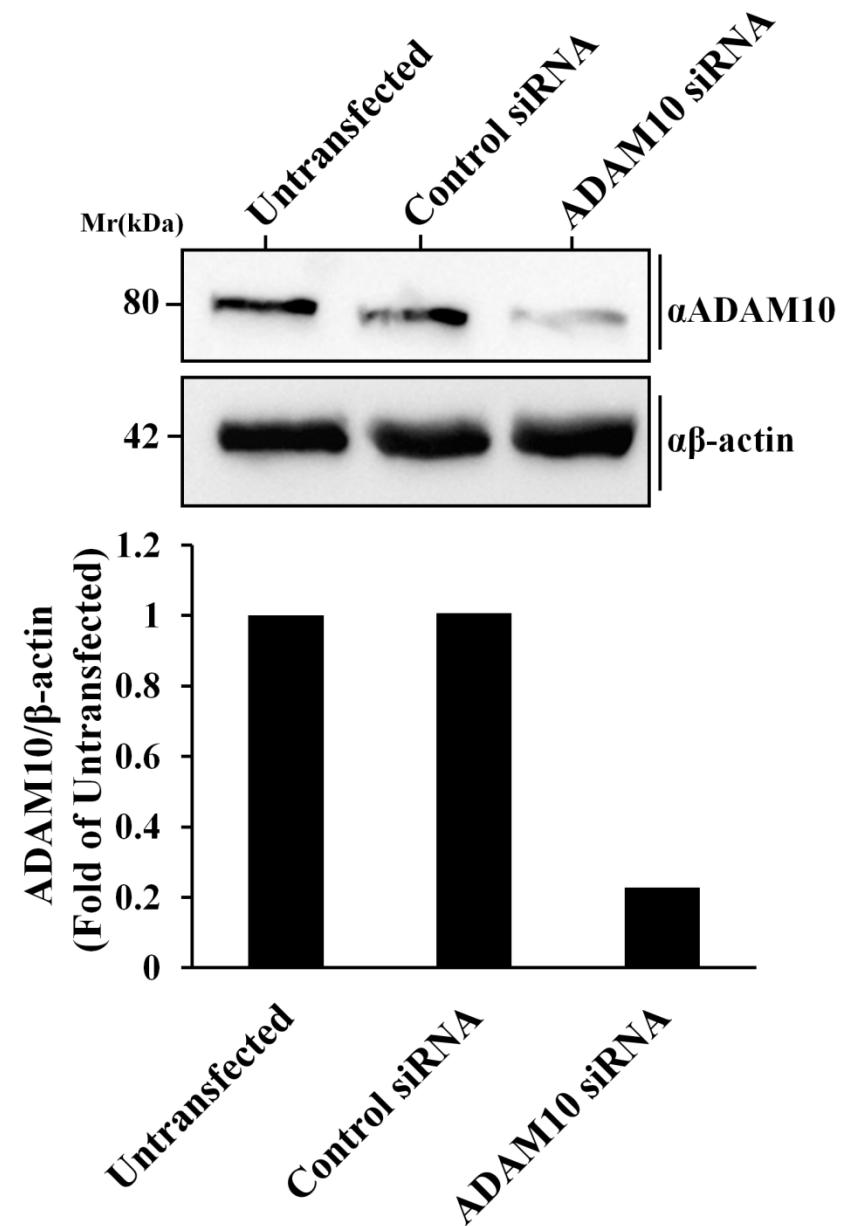

**Fig. S1**

**D**

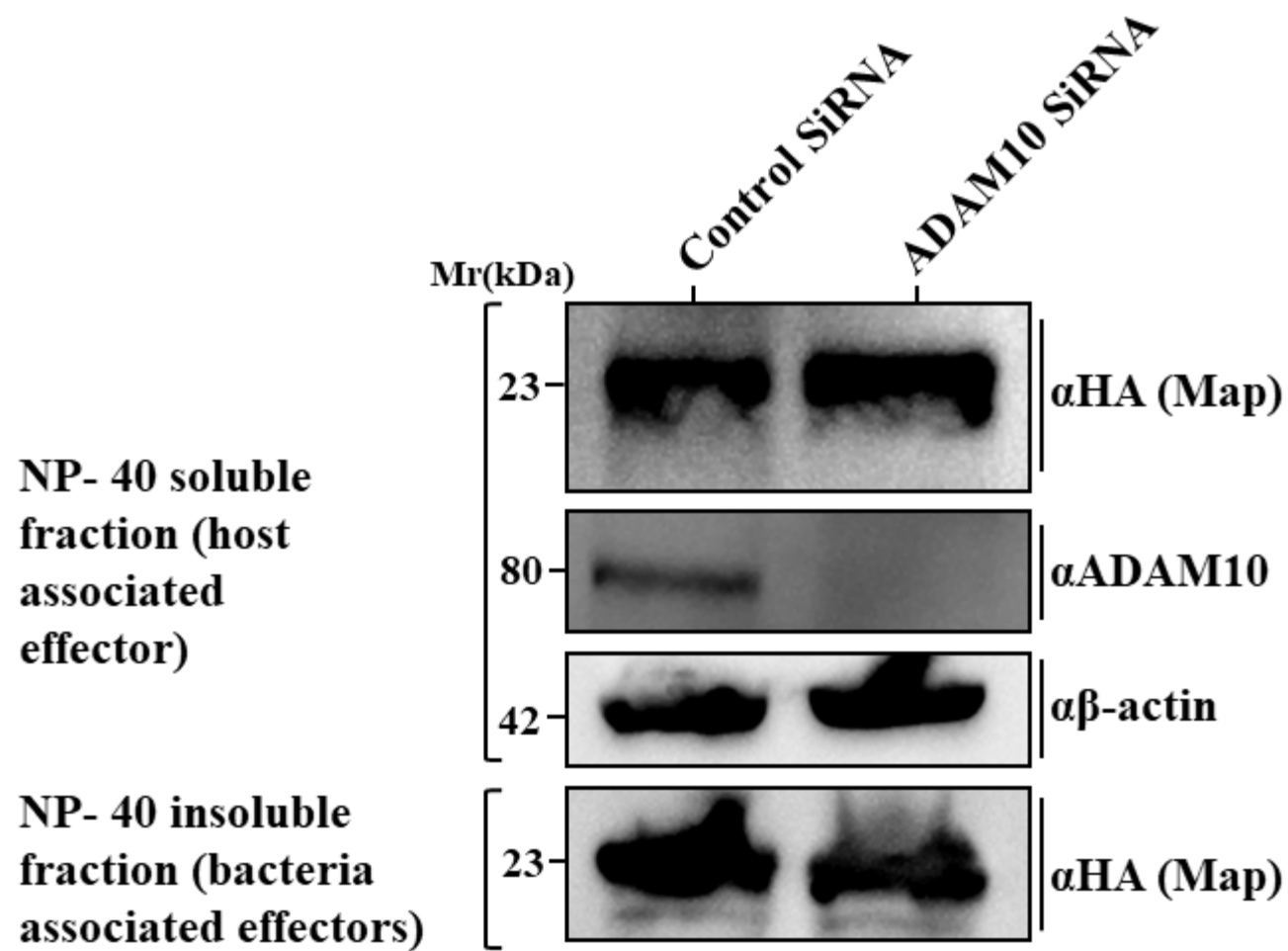

**Fig. S1**

**E**

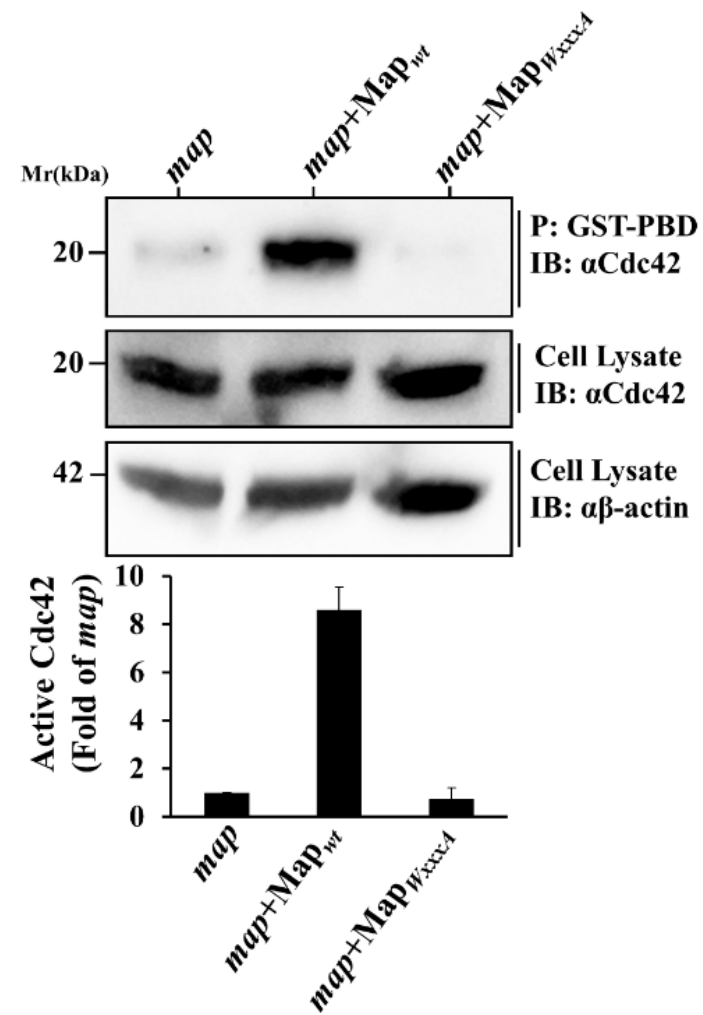

**Fig. S1****F**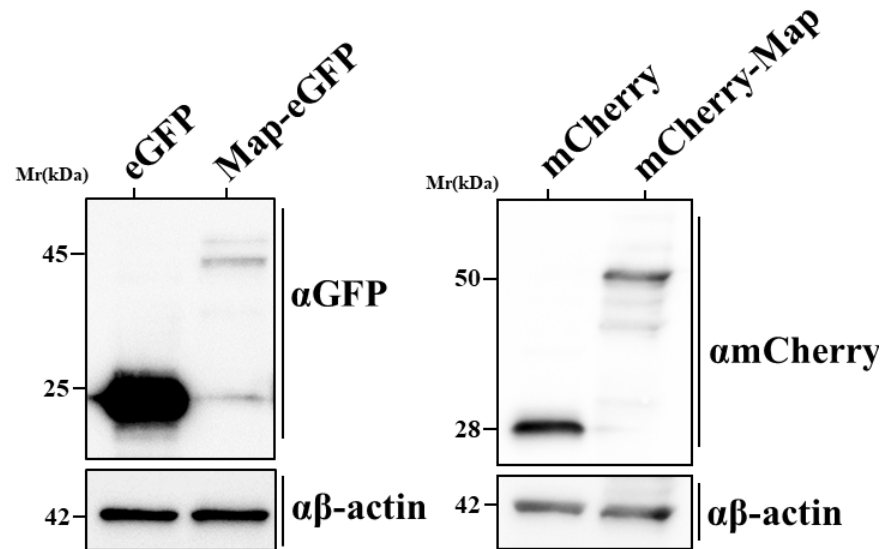**G**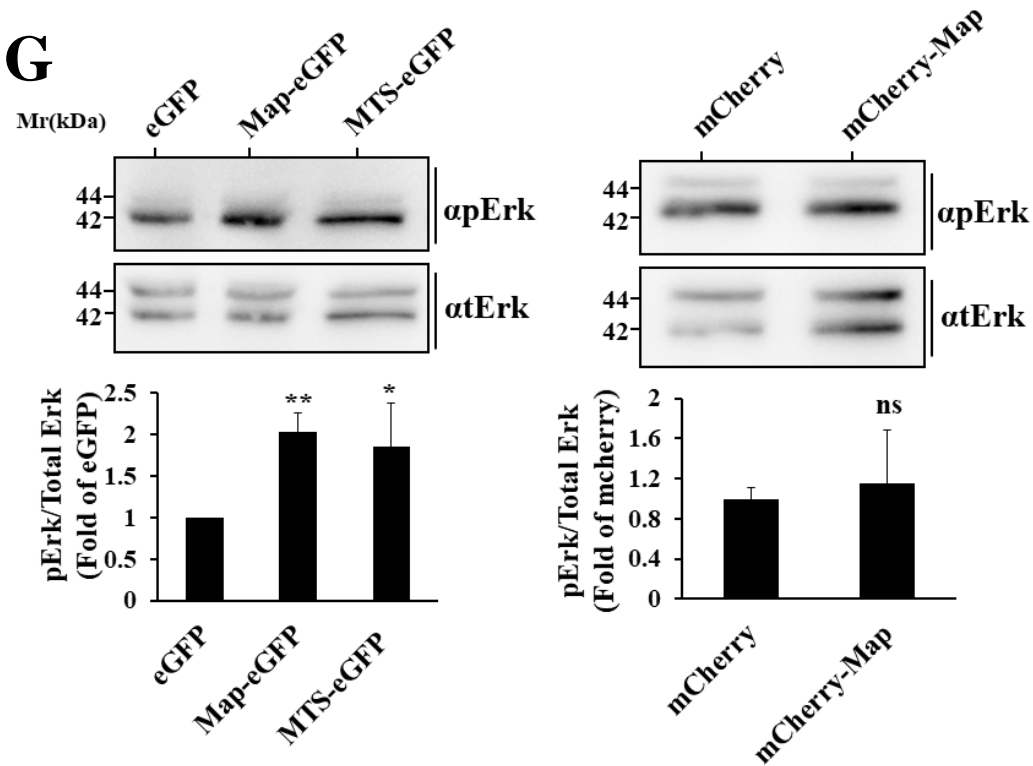**H**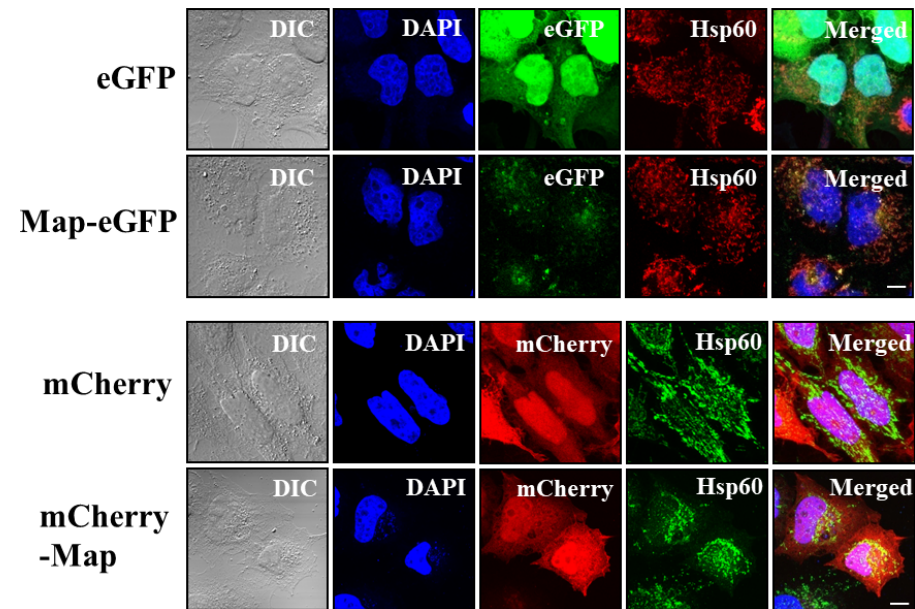

**Fig. S1**

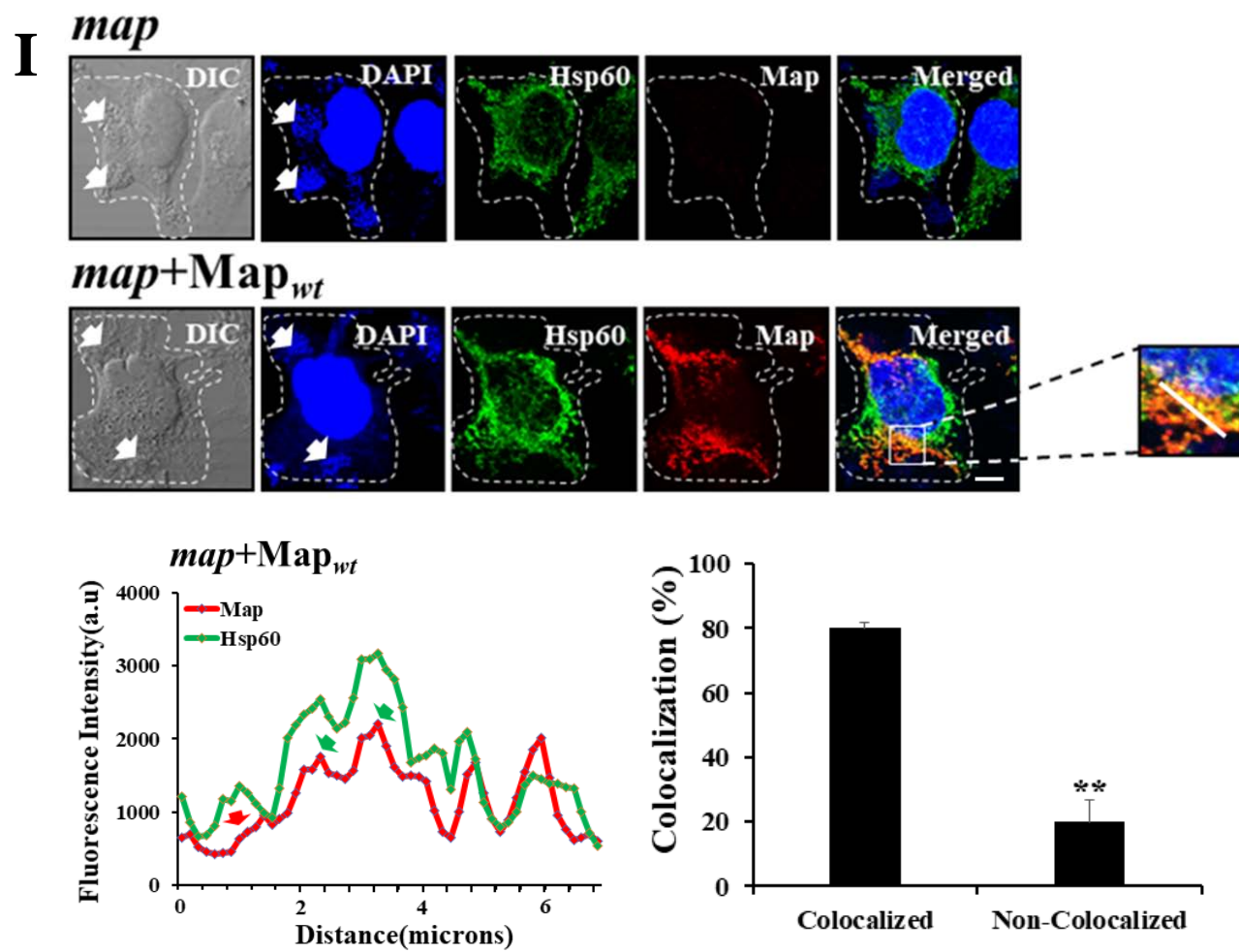

**Fig. S1**

**J**

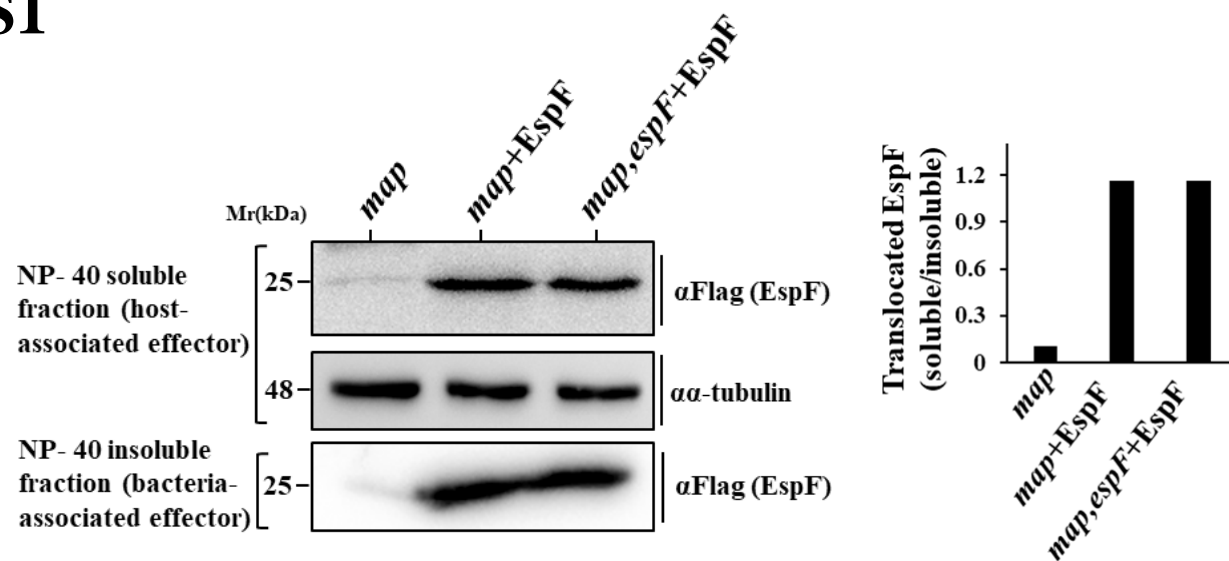

**K**

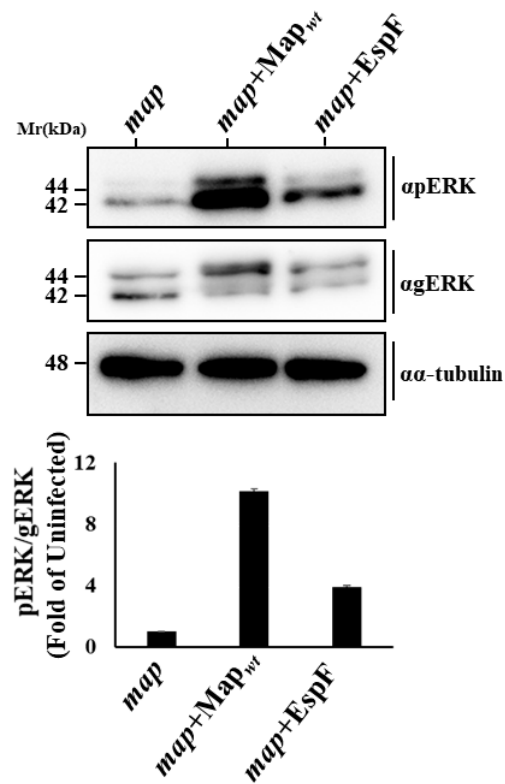

**L**

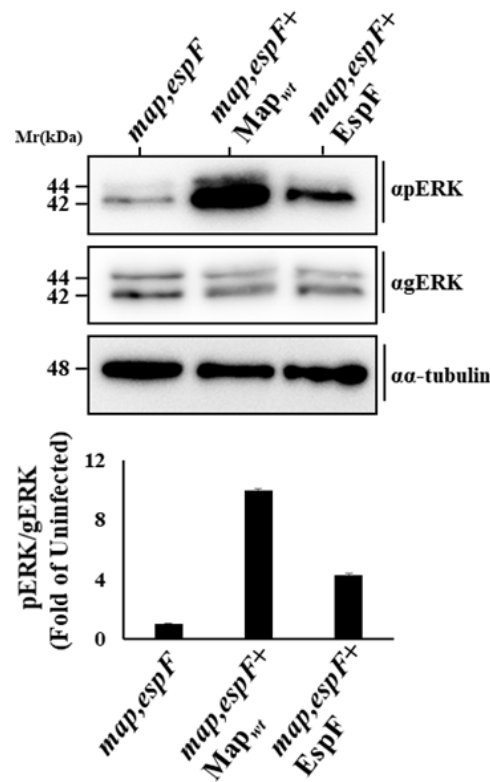

**M**

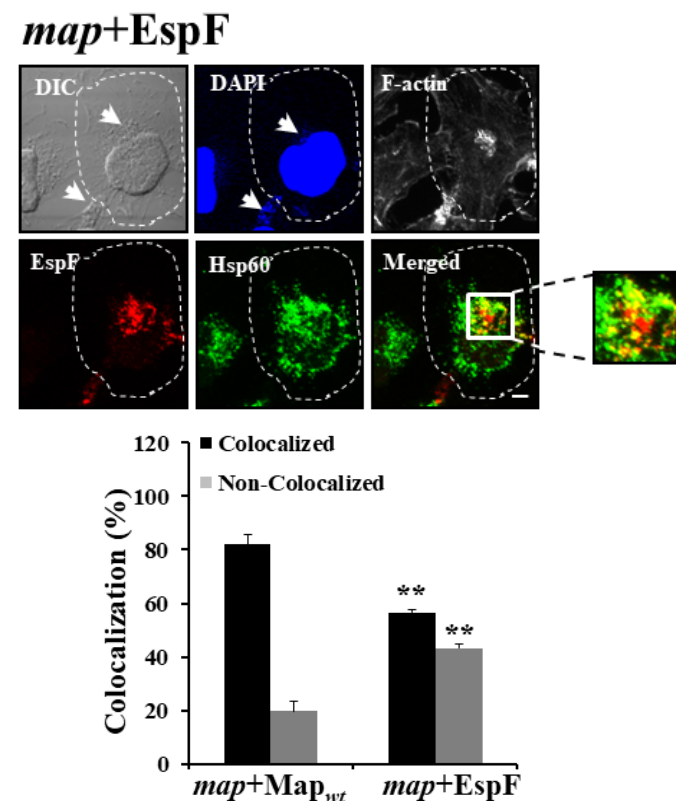

Supplement: FIG S1 [file mBio.01397-20-sf001.pdf]
