# Supplementary material for: Mitochondrial Targeting of the Enteropathogenic Escherichia coli Map Triggers Calcium Mobilization, ADAM10-MAP Kinase Signaling, and Host Cell Apoptosis
Source: mBio. 2020 Sep 15;11(5):e01397-20. doi: 10.1128/mBio.01397-20 (PMC7492733; doi:10.1128/mBio.01397-20)
Supplement: TABLE S2 [file mBio.01397-20-st002.docx]

**Table S2: Primary and secondary antibodies**

| **Antibody** | **Primary/Secondary**  **dilution (IF/WB)** | **Definition/Description** |
| --- | --- | --- |
| Rabbit anti-Phospho (p)- ERK1/2 | Primary  1:2000 (WB) | αpERK; Phospho-p44/42 MAPK (Thr202/Tyr204), #4370; Cell Signaling Technology |
| Rabbit anti-general (g)ERK1/2 | Primary  1:1000 (WB) | αgERK; p44/42 MAPK; #9102; Cell Signaling Technology |
| Rabbit anti-Phospho (p)-MEK1/2 | Primary  1:1000 (WB) | αpMEK; Phospho-MEK1/2 (Ser217/221); #9154; Cell Signaling Technology |
| Rabbit anti-general (g)MEK1/2 | Primary  1:1000 (WB) | αgMEK; MEK1/2 (D1A5); #8727; Cell Signaling Technology |
| Rabbit anti-  Phospho (p)-B-Raf | Primary  1:1000 (WB) | αpB-Raf; Phospho-B-Raf (Ser445); #2696; Cell Signaling Technology |
| Rabbit anti- general (g)B-Raf | Primary  1:1000 (WB) | αgB-Raf; B-Raf (D9T6S) antibody; #14814; Cell Signaling Technology |
| Rabbit anti-Phospho (p)-C-Raf (Ser289/296/301) | Primary  1:1000 (WB) | αpC-Raf; Phospho-C-Raf (Ser289/296/301); #9431; Cell Signaling Technology |
| Rabbit anti-Phospho (p)- C-Raf (Ser338) | Primary  1:1000 (WB) | αpC-Raf; Phospho-C-Raf (Ser338) (56A6); #9427; Cell Signaling Technology |
| Rabbit anti-general (g)C-Raf | Primary  1:1000 (WB) | αgC-Raf; C-Raf antibody; #9422; Cell Signaling Technology |
| Rabbit anti- Phospho EGFR | Primary  1:1000 (WB) | αpEGFR; Phospho-EGF Receptor (Y1068); #2234; Cell Signaling Technology |
| Rabbit anti- EGFR | Primary  1:1000 (WB) | αgEGFR; EGF Receptor (D38B1) antibody; #4267; Cell Signaling Technology |
| Rabbit anti-Phospho (p)- p38α | Primary  1:2000 (WB) | αp-p38; Phospho- αp38 MAPK (Thr180/Tyr182); #4511; Cell Signaling Technology |
| Rabbit anti- general(g) p38α | Primary  1:2000 (WB) | αg-p38; αP38 (C-20); sc-535; Santa Cruz Biotechnology |
| Mouse anti-HA  Tag Mouse  monoclonal  antibody (clone  12CA5) | Primary  1:500 (WB) | αHA; Mouse monoclonal antibody (clone 12CA5)  directed against the 9-amino acid sequence  derived from the influenza hemagglutinin (HA)  protein; #ab16918; Abcam |
| Mouse anti-α-tubulin | Primary  1:2000 (WB) | αα-tubulin; Monoclonal anti-Tubulin-α antibody, Clone B512; T6074; Sigma-Aldrich |
| Mouse anti –β-actin | Primary  1:5000 (WB) | αβ-actin; Monoclonal antibody, ACTN05 (C4);ab3280; Abcam |
| Rabbit anti- ADAM10 | Primary  1:2000 (WB) | αADAM10; Monoclonal anti-ADAM10 antibody [EPR5622] ; ab124695; Abcam |
| Mouse anti-Hsp60 | Primary  1:500 (IF) | αHsp60; Monoclonal anti-Hsp60 antibody; #681502, BioLegend |
| Rabbit anti-Hsp60 | Primary  1:800 (IF) | αHsp60 (D6F1) XP, Monoclonal anti-Hsp60 antibody; #12165, Cell Signaling Technology |
| Mouse anti-Cdc42 | Primary  1:250 (WB) | αCdc42; Monoclonal anti-Cdc42 Clone44(RUO); 610929, BD Transduction Laboratories |
| Mouse anti-GFP | Primary  1:500 (WB) | αGFP; Monoclonal anti-GFP (B-2); sc-9996; Santa Cruz Biotechnology |
| Rabbit anti-mCherry | Primary  1:200 (WB) | mCherry; Polyclonal anti-mCherry antibody ;#AHP2326; Bio-RAD |
| Rabbit anti-Flag | Primary  1:200 (WB) | αFlag; affinity purified anti-Flag antibody; #F7425, Sigma-Aldrich |
| Goat anti-mouse IgG, Alexa Fluor 488 | Secondary  1:300 (IF) | Alexa Fluor 488- AffiniPure Goat anti-Mouse IgG; #115-545-062; Jackson ImmunoResearch Laboratories |
| Peroxidase goat anti-mouse IgG | Secondary  1:10000 (WB) | Peroxidase- AffiniPure Goat anti-Mouse IgG; #115-035-166; Jackson ImmunoResearch Laboratories |
| Peroxidase goat anti-rabbit IgG | Secondary  1:10000 (WB) | Peroxidase- AffiniPure Goat Anti-Rabbit IgG; #111-035-003; Jackson ImmunoResearch Laboratories |
