# Supplementary material for: Mitochondrial Targeting of the Enteropathogenic Escherichia coli Map Triggers Calcium Mobilization, ADAM10-MAP Kinase Signaling, and Host Cell Apoptosis
Source: mBio. 2020 Sep 15;11(5):e01397-20. doi: 10.1128/mBio.01397-20 (PMC7492733; doi:10.1128/mBio.01397-20)
Supplement: TABLE S3 [file mBio.01397-20-st003.docx]

**Table S3: Plasmids**

| **Plasmid name** | **Description** | **Complemented EPEC strain**  **and reference** |
| --- | --- | --- |
| pSA10 | Bacterial expression IPTG inducible vector, Amp^r^ | (1) |
| pKD46 | λ RED genes, Amp^r^ | (2) |
| peGFP-N1 | eGFP-encoding mammalian expression vector | CloneTech Laboratories #6085-1 |
| Map-eGFP | C-terminus of Map tagged with eGFP subcloned into the peGFP-N1 vector | This study |
| mCherry-C1 | mCherry-encoding mammalian expression vector | CloneTech Laboratories  #632524 |
| mCherry-Map | N-terminus of Map tagged with mCherry-C1 | (3) |
| pKD3 | Template for the chloramphenicol resistance cassette, Cam^r^ | (2) |
| pSA10-Map*_wt_* | pSA10 derivative encoding C-terminally HA-tagged Map (aa 1-203) (Map-HA; EPEC 0127:H6 E2348/69) | (4) |
| pSA10-Map_∆_*_MTS_* | pSA10 derivative encoding Map-HA bearing an in-frame deletion (aa 2 - 44) of the N-terminal mitochondrial targeting signal MTS (MTS) (5) | This study |
| pSA10-Map_∆_*_MTS-_*EspH_1-25_ | pSA10 derivative encoding the N-terminal 2-25 amino acids of EspH fused to the N-terminus of Map_∆_*_MTS_* | pSA10-Map_∆_*_MTS_* |
| pSA10-Map*_WxxxA_* | pSA10 derivative encoding Map-HA in which E78 has been substituted for A to generate an inactivated Rho GEF domain (WxxxA) (5) | This study |
| pSA10-Map_∆_*_TRL_* | pSA10 derivative encoding Map-HA in which the C-terminal TRL (201-203) PDZ type I binding motif was deleted in-frame (5) | This study |
| pSA10-Map***_∆_****_101-152_* | pSA10 derivative encoding Map-HA in which amino acids 101-152 were deleted in-frame (5) | This study |
| TGF-α-AP | TGF-α inserted into 3’ end of human alkaline phosphatase (AP) cDNA on a pRc/CMV based expression vector | (6) |
| BTC-AP | Betacellulin inserted into 3’ end of human AP cDNA on a pRc/CMV based expression vector | (6) |

References

1. Schlosser-Silverman E, Elgrably-Weiss M, Rosenshine I, Kohen R, Altuvia S. 2000. Characterization of Escherichia coli DNA lesions generated within J774 macrophages. J Bacteriol 182:5225-30.

2. Datsenko KA, Wanner BL. 2000. One-step inactivation of chromosomal genes in Escherichia coli K-12 using PCR products. Proc Natl Acad Sci U S A 97:6640-5.

3. Litvak Y, Sharon S, Hyams M, Zhang L, Kobi S, Katsowich N, Dishon S, Nussbaum G, Dong N, Shao F, Rosenshine I. 2017. Epithelial cells detect functional type III secretion system of enteropathogenic Escherichia coli through a novel NF-κB signaling pathway. PLoS Pathogens 13:e1006472-e1006472.

4. Berger CN, Crepin VF, Baruch K, Mousnier A, Rosenshine I, Frankel G. 2012. EspZ of enteropathogenic and enterohemorrhagic Escherichia coli regulates type III secretion system protein translocation. MBio 3:00317-12.

5. Dean P, Kenny B. 2009. The effector repertoire of enteropathogenic E. coli: ganging up on the host cell. Curr Opin Microbiol 12:101-9.

6. Sahin U, Weskamp G, Kelly K, Zhou HM, Higashiyama S, Peschon J, Hartmann D, Saftig P, Blobel CP. 2004. Distinct roles for ADAM10 and ADAM17 in ectodomain shedding of six EGFR ligands. J Cell Biol 164:769-79.
