# Supplementary material for: Mitochondrial Targeting of the Enteropathogenic Escherichia coli Map Triggers Calcium Mobilization, ADAM10-MAP Kinase Signaling, and Host Cell Apoptosis
Source: mBio. 2020 Sep 15;11(5):e01397-20. doi: 10.1128/mBio.01397-20 (PMC7492733; doi:10.1128/mBio.01397-20)
Supplement: TABLE S4 [file mBio.01397-20-st004.docx]

**Table S4. List of primers and their usage**

| **Primer name and number** | **Sequence** | **Usage** |
| --- | --- | --- |
| 1354 | GCAATGGTAGGTAGAGCGTTAGCTCAGGCGGTTACACAAACTCTTAGACCCGTGTAGGCTGGAGCTGCTTC | construction of RP8153. |
| 1355 | CTGCAATCGCCTTCTCAGTTAGAGCCTTGATATCACTGATTTTCGCGGTGCCATATGAATATCCTCCTTAG | construction of RP8153. |
| 1371 | GTATCCACTCATGACCATCG | construction of RP8153. |
| 1495 | CTCTGGAATCGACAGAATCAGCC | construction of RP8153. |
| 4197 | GAGCTAACGCTCTACCTACC | construction of RP8153. |
| 4198 | CTAACTGAGAAGGCGATTGC | construction of RP8153. |
| 115 | GATCTTCCGTCACAGGTAGG | verification of RP8153. |
| 1682 | GCTAAACCAGCAGCAATTGCG | verification of RP8153. |
| 1354 | GCAATGGTAGGTAGAGCGTTAGCTCAGGCGGTTACACAAACTCTTAGACCCGTGTAGGCTGGAGCTGCTTC | construction of RP8153. |
| MTS delta For | CATGAATTCTGTTTCCTGTGTGAAATTG | construction of *map*+Map_∆_*_MTS_* |
| MTS delta Rev | TCGAACCTTATGATTAATCATGGCAAAC | construction of *map*+Map_∆_*_MTS_* |
| WxxxA For | AAGCAAGCGCAGATTACTTTTCTATC | construction of *map*+Map*_WxxxA_* |
| WxxxA Rev | GAACGCTTGCTGGGTATCACTAC | construction of *map*+Map*_WxxxA_* |
| Map For | CGTTACTGGTTTCACATTCACC | construction of *map*+Map_∆_*_TRL_* |
| Map Rev | CGCCCACATTGTCTGCAATC | construction of *map*+Map_∆_*_TRL_* |
| TRL gblock | GATTGCAGACAATGTGGGCGCAAGCGTAGTCTGGGACGTCATATGGGTAGGAGCACAGGATTAGTCCCCCGGGGATCCGTCGACCTGCAGCCAAGCTTCGACAATTCGCGCGCGAAGGCGAAGCGGCATGCATTTACGTTGACACCATCGAATGGTGCAAAACCTTTCGCGGTATGGCATGATAGCGCCCGGAAGAGAGTCAATTCAGGGTGGTGAATGTGAAACCAGTAACG | construction of *map*+Map_∆_*_TRL_* |
| ∆MTS Linear F | TCGAACCTTATGATTAATCATGG | Construction of *map*+Map_∆_*_MTS-_*EspH_1-25_ |
| ∆MTS Linear R | GAATTCTGTTTCCTGTGTGAAAT | Construction of *map*+Map_∆_*_MTS-_*EspH_1-25_ |
| EspH 1-25aa gblock | CAATTTCACACAGGAAACAGAATTCATGAGCAGCAGCCTGAGCGGCATTACCTTTACCACCAGCCTGACCAGCCATGCGAGCTGGAACAAACTGACCCGCTCGAACCTTATGATTAATCATGGCAAAC | Construction of *map*+Map_∆_*_MTS-_*EspH_1-25_ |
| Map delta 101-152 Linear F | CTTTGAACAGAATTTTAGCAC | Construction of *map*+Map*_∆101-152_* |
| Map delta 101-152 Linear R | GCCATGATTAATCATAAGGTT | Construction of *map*+Map*_∆101-152_* |
| Delta 101-152 gblock | CCTTATGATTAATCATGGCAAACTGACTACCCAGCTACTACAGGCCGTAGCCAAACAAACTGGTAGTAGTGATACCCAGCAATGGTTCAAGCAAGAGCAGATTACTTTTCTATCCAGAGCAGTAAACAAAACTGTGGATGACTATTGCATGAGTAATAATTCGGTGGATCCCATTACACGTTTTAACACTCAAACCAAATTGATAGAAGTCTCACGCGAAATCTTTGAACAGAATTTTA | Construction of *map*+Map*_∆101-152_* |
| Map F seq | CGCACTCCCGTTCTGGATAATG | verification of *map*+Map*_∆MTS_*, *map*+Map*_∆TRL_*, map+Map*_WxxxA,_ map*+Map_∆_*_MTS-_*EspH_1-25_ *and map*+Map*_∆101-152_* |
| Map R seq LacI | CGTTACTGGTTTCACATTCACC | verification of *map*+Map*_∆MTS_*, *map*+Map*_∆TRL_*, *map*+Map*_WxxxA,_ map*+Map_∆_*_MTS-_*EspH_1-25_ *and map*+Map*_∆101-152_* |
| Map EGFP For | AGTGAACCGTCAGATCCATGTTTAGTCCAACGGCAATG | to produce Map insert with eGFP overhangs from pSA10-Map for the creation of Map-eGFP |
| Map EGFP Rev | GCTCACCATGGTGGCGCAGCCGAGTATCCTGCACA | to produce Map insert with eGFP overhangs from pSA10-Map for the creation of Map-eGFP |
| pEEF7 | CGCCAATAGGGACTTTCCAT | verification of Map-eGFP |
